# Cationic cobalt-catalyzed [1,3]-rearrangement of *N*-alkoxycarbonyloxyanilines

**DOI:** 10.3762/bjoc.14.172

**Published:** 2018-07-31

**Authors:** Itaru Nakamura, Mao Owada, Takeru Jo, Masahiro Terada

**Affiliations:** 1 Research and Analytical Center for Giant Molecule, Graduate School of Science, Tohoku University, 6-3 Aramaki Aza Aoba, Aoba-ku, Sendai 980-8578 Japan; 2 Department of Chemistry, Graduate School of Science, Tohoku University, 6-3 Aramaki Aza Aoba, Aoba-ku, Sendai 980-8578 Japan

**Keywords:** anilines, cobalt catalyst, concerted reaction, N–O bond, rearrangement

## Abstract

A cationic cobalt catalyst efficiently promoted the reaction of *N*-alkoxycarbonyloxyanilines at 30 °C, affording the corresponding *ortho*-aminophenols in good to high yields. As reported previously, our mechanistic studies including oxygen-18 labelling experiments indicate that the rearrangement of the alkoxycarbonyloxy group proceeds in [1,3]-manner. In this article, we discuss the overall picture of the cobalt-catalysed [1,3]-rearrangement reaction including details of the reaction conditions and substrate scope.

## Introduction

**Scheme 1:**
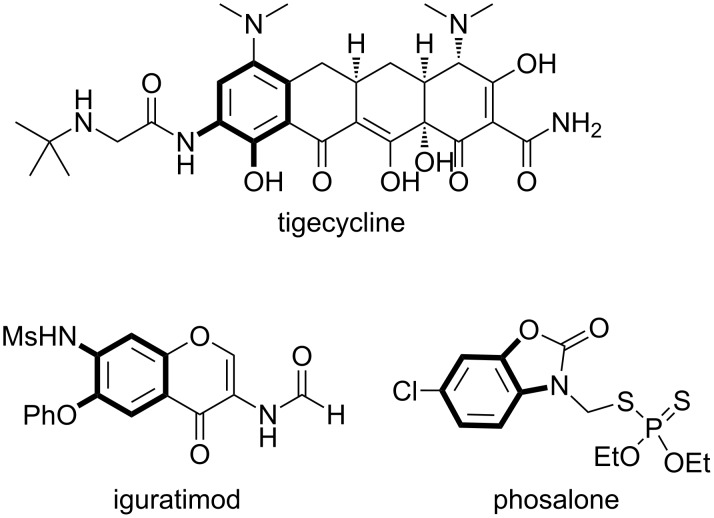
*ortho*-Aminophenol derivatives.

**Scheme 2:**
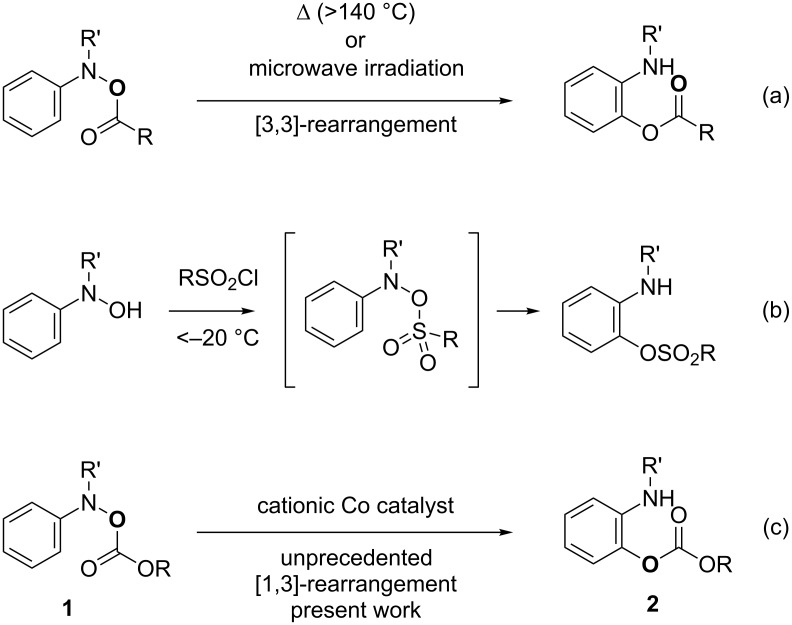
Rearrangement of *N*-acyloxyanilines.

The 2-aminophenol moiety is ubiquitously found as a core structure of biologically active compounds, such as tigecycline [1], iguratimod [2], and phosalone (Scheme 1) [3]. The scaffolds have also been frequently utilized as synthetic intermediates not only in pharmaceutical chemistry but also in materials science. Thus, it is of great importance to efficiently synthesize functionalized 2-aminophenols under mild reaction conditions in a regioselective manner. Among numerous methods, the [3,3]-rearrangement of *O*-acyl-*N*-arylhydroxylamines **1** driven by cleavage of the N–O bond is an ideal approach to selectively synthesize *O*-protected 2-aminophenols **2** while maintaining the oxidation state during the transformation (Scheme 2a) [4–11]. However, there is a significant drawback, these [3,3]-rearrangements of carboxylic acyloxy and alkoxylcarbonyloxy groups generally require long heating times at elevated reaction temperatures (>140 °C) or microwave irradiation (Scheme 2a). In contrast, *N*-sulfonyloxyanilines are known to readily undergo the [3,3]-rearrangement during the preparation of the starting material below −20 °C due to the strongly electron-withdrawing nature of the sulfonyl group (Scheme 2b) [12]. Accordingly, we envisioned that appropriate Lewis acidic metal catalysts would promote the rearrangement reaction of stable *N*-acyloxyanilines to afford readily deprotectable 2-acyloxyanilines under much milder reaction conditions with high functional group tolerance. Based on this concept, we disclosed that cationic cobalt catalysts efficiently promote the reaction of O-alkoxylcarbonyl-*N*-arylhydroxylamines **1** at 30 °C, affording the corresponding 2-aminophenol derivatives **2** in good to high yields [13]. Our mechanistic studies revealed that the rearrangement of the alkoxycarbonyloxy group proceeded in an unprecedented [1,3]-manner (Scheme 2c). In this article, we describe the overall picture of the intriguing [1,3]-rearrangement reaction, particularly the detail of the reaction, which were not sufficiently discussed in our preliminary communication.

## Results and Discussion

At the beginning of this investigation, *N,O-*di(methoxycarbonyl)hydroxylaniline (**1a**) was treated with catalytic amounts of several copper salts in 1,2-dichloroethane (DCE) at 60 °C (Table 1, entries 1–7), according to our previous copper-catalysed cascade reaction involving rearrangement via N–O bond cleavage [14]. While divalent copper acetate and copper chloride did not show any catalytic activities (Table 1, entries 1 and 2), more Lewis acidic copper complexes, such as [Cu(MeCN)_4_](PF_6_)_2_ and [Cu(OTf)]_2_·toluene, afforded the corresponding 2-aminophenol derivative **2a** (Table 1, entries 3 and 4). Moreover, a cationic copper catalyst generated from CuCl_2_ and two equivalents of AgSbF_6_ was effective to afford **2a** in good yield (Table 1, entry 5), even at 30 °C (Table 1, entry 8). The use of a ligand, such as 1,10-phenanthroline (phen) and 1,3-bis(diphenylphosphino)propane (dppp), totally diminished the activity of cationic cobalt catalyst (Table 1, entries 6 and 7). Among metal chlorides examined, CoCl_2_ exhibited the best catalytic activity at 30 °C, affording the corresponding **2a** in 68% yield (Table 1, entry 9), as reported previously [13]. A divalent cationic zinc catalyst also promoted the present reaction, albeit with lower chemical yield than Co(II) (Table 1, entries 10 and 11), while the use of Fe(II) and Pd(II) resulted in low chemical yield due to the formation of the *para*-isomer **3a** (Table 1, entries 12 and 13). Indeed the *para*-isomer **3a** was obtained as a major product when the reaction of **1a** was conducted using trivalent metal salts, such as FeCl_3_ and RuCl_3_, and tetravalent salts, such as ZrCl_4_, as a catalyst (Table 1, entries 15–18). Although we quite recently disclosed that cationic NHC-copper catalysts efficiently promoted the [1,3]-alkoxy rearrangement of *N*-alkoxyaniline [15], the cationic NHC-Cu catalyst generated from IPrCuBr and AgSbF_6_ was totally inefficient for the present reaction; **1a** was decomposed under the reaction conditions (Table 1, entry 19). Whereas AgSbF_6_ promoted the reaction at 60 °C (Table 1, entry 20), the catalytic activity was diminished at 30 °C (Table 1, entry 21). Neutral CoCl_2_ did not promote the present reaction (Table 1, entry 22). Brønsted acids, such as trifluoromethanesulfonic acid and diphenylphosphoric acid, were much less active (Table 1, entries 23 and 24). The kind of the counteranion significantly affected the reaction efficiency; hexafluoroantimonate and bis(trifluoromethanesulfonyl)imidate were efficient (Table 1, entries 9 and 25), while the use of hexafluorophosphate and trifluoromethanesulfonate did not promote the reaction at all (Table 1, entries 26 and 27). The use of an equal amount of AgSbF_6_ to CoCl_2_ resulted in slightly decreasing the chemical yield (Table 1, entry 28).

**Table 1:** Catalytic activity.

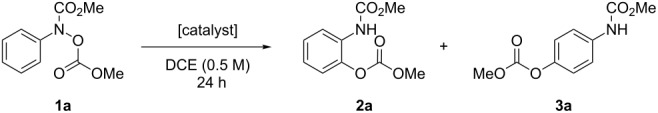					

entry	catalyst (mol %)	temp. (°C)	**2a** (%)^a^	**3a** (%)^a^	**1a** (%)^a^

1	CuCl_2_ (10)	60	<1	<1	>99
2	Cu(OAc)_2_ (10)	60	<1	<1	>99
3	[Cu(MeCN)_4_](PF_6_) (10)	60	5	<1	75
4	[Cu(OTf)]_2_·C_6_H_5_CH_3_ (10)	60	50	4	23
5	CuCl_2_ (10), AgSbF_6_ (20)	60	52	<1	14
6	CuCl_2_ (10), AgSbF_6_ (20), phen (20)	60	<1	<1	>99
7	CuCl_2_ (10), AgSbF_6_ (20), dppp (20)	60	<1	<1	80
8^b^	CuCl_2_ (10), AgSbF_6_ (20)	30	57	10	<1
9^b^	CoCl_2_ (10), AgSbF_6_ (20)	30	63 (68)	<3	<1
10^b^	ZnCl_2_ (10), AgSbF_6_ (20)	30	54	8	<1
11	ZnCl_2_ (10), AgSbF_6_ (10)	30	54	8	1
12^b^	PdCl_2_ (10), AgSbF_6_ (20)	30	24	7	<1
13^b^	FeCl_2_ (10), AgSbF_6_ (20)	30	32	21	1
14^b^	FeCl_3_ (10), AgSbF_6_ (30)	30	22	26	<1
15^b^	RuCl_3_ (10), AgSbF_6_ (30)	30	18	35	<1
16	RuCl_3_ (10), AgSbF_6_ (20)	30	21	35	<1
17	IrCl_3_ (10), AgSbF_6_ (30)	30	15	37	<1
18	ZrCl_4_ (10), AgSbF_6_ (40)	30	16	28	<1
19	IPrCuBr (10), AgSbF_6_ (10)	30	<1	<1	12
20	AgSbF_6_ (10)	60	53	17	7
21^b^	AgSbF_6_ (10)	30	<3	<3	80
22^b^	CoCl_2_ (10)	30	<1	<1	90
23	TfOH (10)	30	5	6	74
24	(PhO)_2_P(O)OH	30	<1	<1	90
25^b^	CoCl_2_ (10), AgNTf_2_ (20)	30	50	1	3
26^b^	CoCl_2_ (10), AgPF_6_ (20)	30	<1	<1	98
27^b^	CoCl_2_ (10), AgOTf (20)	30	<1	<1	97
28	CoCl_2_ (10), AgSbF_6_ (10)	30	53	3	<1
29	CoCl_2_ (5), AgSbF_6_ (10)	30	59	2	<1

^a^Yields were determined by ^1^H NMR using CH_2_Br_2_ as an internal standard. Isolated yield in parenthesis. ^b^Reported in the Supporting Information of our previous paper [13], except for yields of recovered **1a**.

Next solvent and concentration effects were examined as summarized in Table 2. 1,2-Dichloroethane (DCE) gave the best result (Table 2, entry 1), as described previously [13]. Other halogen solvents, such as CHCl_3_, CH_2_Cl_2_, and PhCl, ethereal solvent, such as Et_2_O and *tert*-butyl methyl ether (MTBE), and toluene were less efficient (Table 2, entries 2–7), while the use of polar solvents, such as tetrahydrofuran (THF), acetonitrile, and *N*,*N*-dimethylformamide (DMF), resulted in quantitative recovery of the starting material **1a** (Table 2, entries 8–10). A protic solvent, such as methanol, was ineffective (Table 2, entry 11). Slight dilution of the reaction solution (0.25 M) improved the chemical yield (Table 2, entry 12).

**Table 2:** Solvent and concentration effects.

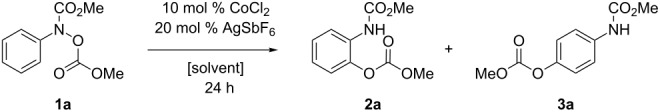					

entry	solvent	concentration (M)	**2a** (%)^a^	**3a** (%)^a^	**1a** (%)^a^

1^b^	DCE	0.5	63	3	<1
2^b^	CHCl_3_	0.5	49	7	19
3^b^	CH_2_Cl_2_	0.5	40	2	<1
4^b^	PhCl	0.5	39	2	25
5^b^	toluene	0.5	43	1	11
6^b^	Et_2_O	0.5	38	<1	1
7	MTBE	0.5	49	1	4
8	THF	0.5	<1	<1	>99
9	CH_3_CN	0.5	<1	<1	98
10	DMF	0.5	<1	<1	>99
11	MeOH	0.5	4	<1	81
12	DCE	1.0	51	2	<1
13^b^	**DCE**	**0.25**	**72**	**3**	**<1**
14^b,c^	DCE	0.05	53	9	11

^a^Yields were determined by ^1^H NMR using CH_2_Br_2_ as an internal standard. ^b^Reported in the Supporting Information of our previous paper (ref. [13]), except for yields of recovered **1a**. ^c^For 5 days.

As mentioned previously [13], carbamate-type groups, such as methoxycarbonyl, Alloc and Cbz were tolerated as a protective group on the nitrogen atom, affording the desired products **2** in good yields (Table 3, entries 1–3). The reaction of **1e** having a 2,2,2-trichloroethoxycarbonyl (Troc) group, however, resulted in decomposing **1e** (Table 3, entry 4). The use of aroyl groups gave the desired product in good yields (Table 3, entries 5–7), while the acetyl group required a prolonged reaction time (Table 3, entry 8). Substrate **1j** having a tosyl group on the nitrogen resulted in decomposition of **1j** (Table 3, entry 9). The alkoxycarbonyl groups, such as Cbz, methoxycarbonyl, and 2-chloroethoxy groups, were employed as good migrating groups (Table 3, entries 7–11), while **1m** having a Boc group on the oxygen atom did not give the desired product, due to decomposition of **1m** (Table 3, entry 12). It is noteworthy that the substrate having a highly electron-withdrawing Troc group on the oxygen atom was readily isomerized to the *ortho*-aminophenol derivative under its preparing conditions in the absence of the cationic cobalt catalyst. In sharp contrast to alkoxycarbonyloxy groups, acyloxy groups, such as the benzoyloxy group, were not migrated to the *ortho*-position, resulting in decomposing the starting material (Table 3, entry 13), as mentioned previously [13].

**Table 3:** Substituent effect at the hydroxylamine moiety.^a^

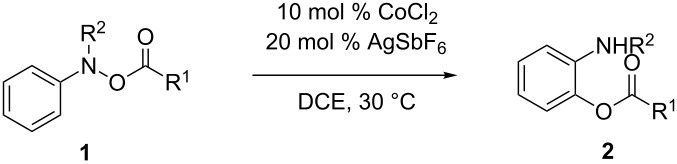						

entry	**1**	R^1^	R^2^	time (h)	**2**	yield (%)^b^

1^c^	**1b**	OMe	Cbz	5	**2b**	64
2^c^	**1c**	OMe	Alloc	4	**2c**	45
3^c^	**1d**	OMe	Boc	6	**2d**	64
4	**1e**	OMe	Troc	18	–	<1
5^c^	**1f**	OMe	*p*-MeOC_6_H_4_C(O)	2	**2f**	60^d^
6^c^	**1g**	OMe	Bz	3	**2g**	75
7^c^	**1h**	OMe	*p*-F_3_CC_6_H_4_C(O)	24	**2h**	61
8^c^	**1i**	OMe	Ac	120	**2i**	44
9	**1j**	OMe	Ts	11	–	<1
10^c^	**1k**	OBn	Bz	2	**2k**	82
11	**1l**	O-CH_2_CH_2_Cl	Bz	2	**2l**	56
12	**1m**	O-*t*Bu	Bz	10	–	<1
13^c^	**1n**	Ph	Bz	120	–	<1

^a^The reactions of **1** (0.4 mmol) were conducted in the presence of 10 mol % CoCl_2_ and 20 mol % of AgSbF_6_ in DCE (1.6 mL) at 30 °C. ^b^Isolated yield. ^c^Previously reported in [13]. ^d1^H NMR yield using dibromomethane as an internal standard.

The reaction was applied to **1o–ab** having various substituents at the *para*-position, as summarized in Table 4. As reported previously [13], reactive functional groups, such as bromo, iodo, and alkynyl groups were tolerated, affording the desired products in good to high yields (Table 4, entries 5–7). The substrate **1v** having a methoxycarbonyl group afforded **2v** in good yield, while cyano and acetyl groups interrupted the present reaction presumably due to deactivation of the catalyst, recovering the starting materials quantitatively (Table 4, entries 9 and 10). Notably, our catalytic conditions successfully promoted the rearrangement of **1y’**, having a highly electron-deficient *p*-trifluromethylphenyl group, which have not been employed in the thermal [3,3]-rearrangement reaction, when using a *p*-nitrobenzyloxycarbonyl group in place of Cbz as the migrating group (Table 4, entry 11). In addition, compatibility of the protective group on the oxygen atom was tested (Table 4, entries 12–14), since it is expected that the cationic cobalt would make the protective group labile as well as the protective group would deactivate the cationic cobalt catalyst. As results, *tert*-butyldimethylsilyl (TBS) and methoxymethyl (MOM) groups were tolerated under the cationic cobalt-catalyzed reaction conditions to afford the desired product in good yields (Table 4, entries 13 and 14). The reaction using a benzoyl group was sluggish, affording the desired product **2z** in moderate yield with formation of inseparable byproducts (Table 4, entry 12). Thus, the use of silyl- and acetal-type protective groups is suitable for the present reaction.

**Table 4:** Co-catalyzed reaction of *N*-alkoxycarbonyloxyanilines **1o–ab**.^a^

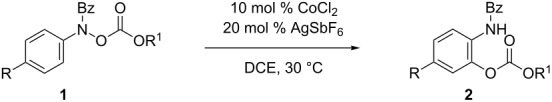						

entry	**1**	R	R^1^	time (h)	**2**	yield (%)^b^

1^c^	**1o**	Me	Bn	3	**2o**	74
2^c^	**1p**	F	Bn	11	**2p**	66^d^
3^c^	**1q**	Cl	Bn	1	**2q**	88
4^c^	**1r**	Cl	Me	3	**2r**	79
5^c^	**1s**	Br	Bn	1	**2s**	86
6^c^	**1t**	I	Bn	1	**2t**	77
7^c^	**1u**	TMSC≡C	Bn	2	**2u**	62
8^c^	**1v**	CO_2_Me	Bn	15	**2v**	84^d^
9	**1w**	Ac	Bn	>120	–	<1^e^
10	**1x**	CN	Bn	>120	–	<1^e^
11^c^	**1y’**	CF_3_	*p*-O_2_NC_6_H_4_CH_2_	48	**2y’**	76^d,f^
12	**1z**	BzO(CH_2_)_2_	Bn	72	**2z**	50^d,g^
13	**1aa**	TBSO(CH_2_)_2_	Bn	14	**2aa**	64
14	**1ab**	MOM(CH_2_)_2_	Bn	14	**2ab**	59

^a^The reactions of **1** (0.4 mmol) were conducted in the presence of 10 mol % CoCl_2_ and 20 mol % of AgSbF_6_ in DCE (1.6 mL) at 30 °C. ^b^Isolated yield. ^c^Previously reported in [13]. ^d1^H NMR yield using dibromomethane as an internal standard. See Supporting Information File 1 for details. ^e^The starting material was quantitatively recovered. ^f^Yield brsm (28% of **1y’** was recovered). ^g^Isolation of **2z** was unsuccessful due to contamination by inseparable byproducts (see Supporting Information File 1).

**Scheme 3:**
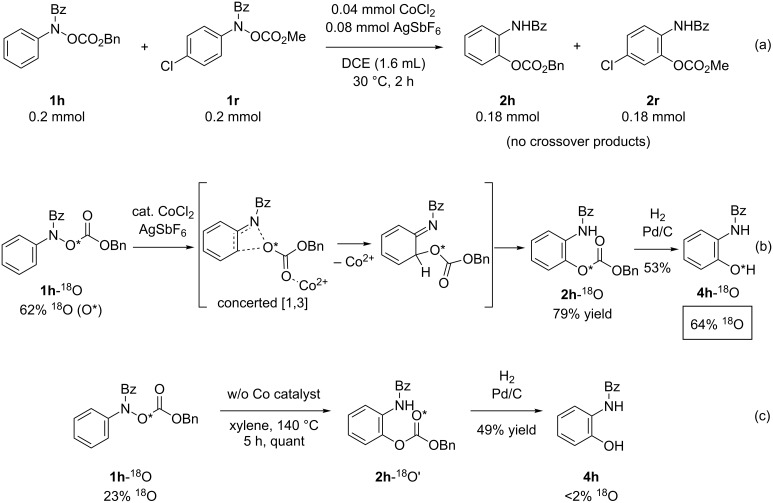
Mechanistic studies, reported in [13].

**Scheme 4:**
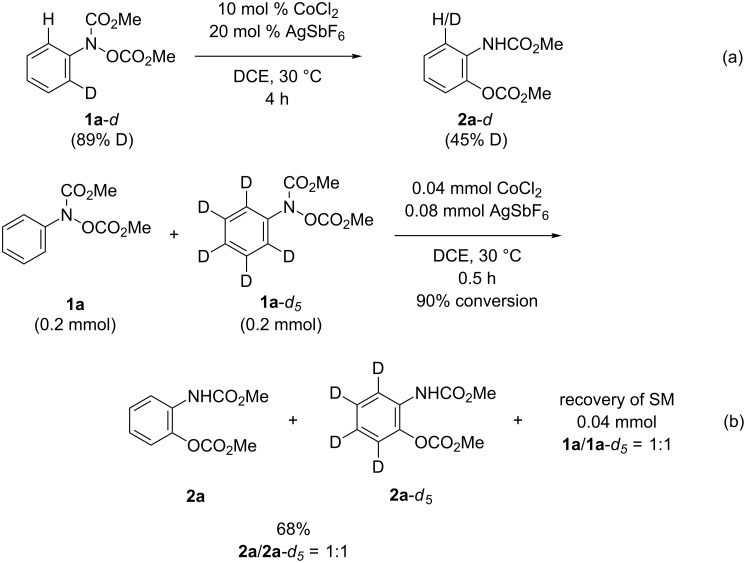
Competitive experiments, reported in [13].

As reported previously [13], the fact that the present rearrangement reaction proceeds in a [1,3]-manner was confirmed by a crossover experiment and oxygen-18 labeling experiments. That is, the reaction of a 1:1 mixture of equally-reactive substrates **1h** and **1r** under the standard reaction conditions afforded only the products **2h** and **2r** derived from the starting materials (Scheme 3a). Thus, we confirmed that the present reaction proceeds in an intramolecular manner. Next, 18-oxygen-labelling experiments were conducted using substrate **1h**-^18^O, of which the oxygen-18 content at the hydroxylamine oxygen atom was 62% [16–17]. The reaction of **1h**-^18^O in the presence of the cationic cobalt catalyst at 30 °C followed by hydrogenative cleavage of the Cbz group afforded the phenol **4h**-^18^O, of which the oxygen-18 content was 64% (Scheme 3b). The result clearly indicates that the rearrangement of the CbzO group in the presence of cationic cobalt catalysts proceeds in a concerted [1,3]-manner [18–24]. In addition, the reaction of **1h**-^18^O (23% ^18^O) in the absence of the cationic cobalt catalyst at 140 °C followed by hydrogenative deprotection afforded **4h**, of which the oxygen-18 content was less than 2% (Scheme 3c). Therefore, we concluded that the cationic cobalt catalyst not only made the reaction much milder than the thermally-induced reaction but also changed the rearrangement mode to an unprecedented [1,3]-manner. In addition, intermolecular and intramolecular competitive experiments using deuterium-labelled substrates resulted in no kinetic effect (Scheme 4). These results suggest that the C–O bond would form prior to cleavage of the C–H bond in the [1,3]-rearrangement reaction.

**Scheme 5:**
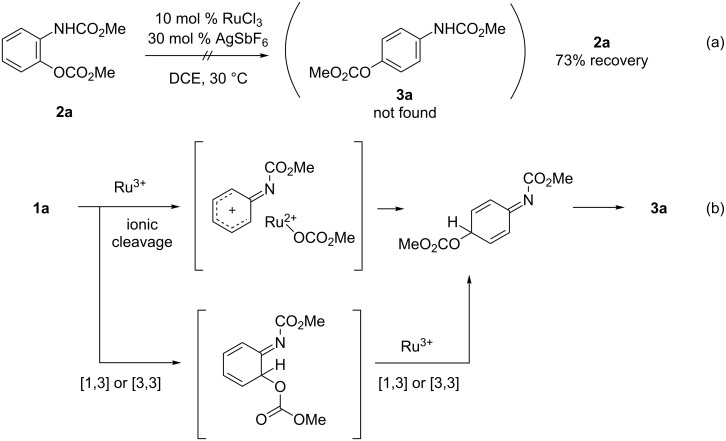
Mechanism for rearrangement to the *para*-position.

Due to the fact that the reaction of **1a** in the presence of tri- and tetravalent cationic metal catalysts afforded the *para*-isomer **3a** as a major product (Table 1, entries 14–18), the reaction of *ortho*-aminophenol derivative **2a** in the presence of catalytic amounts of RuCl_3_ and AgSbF_6_ was conducted. However, the *para*-isomer **3a** was not afforded; 73% of **2a** was recovered (Scheme 5a). The result indicates that the *para*-isomer **3a** was not formed through the *ortho*-isomer **2a**. It is assumed that **3a** was furnished through direct C–O bond formation at the *para*-position through ionic cleavage of the N–O bond by cationic Ru(III) as a much stronger Lewis acid, while it is also possible that the second migration of the alkoxycarbonyloxy group from *ortho* to *para* occurs prior to proton transfer (Scheme 5b) [25]. Further mechanistic studies are underway in our laboratory.

## Conclusion

The cationic cobalt catalysts enabled the rearrangement reaction of *N*-alkoxycarbonyloxyanilines to proceed under much milder reaction conditions, expanding the substrate scope to more electron-deficient anilines. More importantly, the cobalt catalyst changes the mode of the rearrangement to an unprecedented [1,3]-manner.

## Experimental

To a mixture of **1k** (138.9 mg, 0.4 mmol), CoCl_2_ (5.2 mg, 0.04 mmol), and AgSbF_6_ (27.5 mg, 0.08 mmol) under an argon atmosphere in a pressure vial was added 1,2-dichloroethane (1.6 mL). Then, the mixture was stirred at 30 °C for 2 hours. After complete consumption of the starting material **1k**, the mixture was passed through a small pad of silica gel with ethyl acetate. After removing the solvents in vacuo, the residue was purified by flash silica gel column chromatography using hexane/ethyl acetate (3:1) as eluent to obtain **2k** (113.9 mg, 82%).

## Supporting Information

File 1 General procedure and analytic data for obtained products.
