# Supplementary material for: Cationic cobalt-catalyzed [1,3]-rearrangement of N-alkoxycarbonyloxyanilines
Source: Beilstein J Org Chem. 2018 Jul 31;14:1972–9. doi: 10.3762/bjoc.14.172 (PMC6122159; doi:10.3762/bjoc.14.172)
Supplement: File 1 — General procedure and analytic data for obtained products. [file Beilstein_J_Org_Chem-14-1972-s001.pdf]

Supporting Information

for

Cationic cobalt-catalyzed [1,3]-rearrangement of *N*-alkoxycarbonyloxyanilines

Itaru Nakamura[1]*, Mao Owada[2], Takeru Jo[2] and Masahiro Terada[1,2]

Address: [1]Research and Analytical Center for Giant Molecule, Graduate School of Science, Tohoku University, 6-3 Aramaki Aza Aoba, Aoba-ku, Sendai 980-8578 Japan and [2]Department of Chemistry, Graduate School of Science, Tohoku University, 6-3 Aramaki Aza Aoba, Aoba-ku, Sendai 980-8578 Japan

Email: Itaru Nakamura - itaru-n@tohoku.ac.jp

* Corresponding author

General procedure and analytic data for obtained products

1. General information S2
2. Thermally induced reaction of **1y'** S2
3. Crossover experiment (analyzed by HRMS) S3
4. Intramolecular competitive experiments S5
5. ^{18}O-Labeling experiments S7
6. Analytical data of **1** S18
7. Analytical data of **2** S21
8. Analytical data of **3** S23
9. ^{1}H and ^{13}C NMR chart of **1**, **2**, **3,** and **4** S24
10. Reference S37

1. General Information

^{1}H and ^{13}C NMR spectra were recorded on a JEOL JNM-ECS400 (400 MHz for ^{1}H and 100 MHz for ^{13}C) spectrometer and JEOL JNM-ECA600 (150 MHz for ^{13}C)) spectrometer. Chemical shifts are reported in ppm relative to $CHCl_3$ (for ^{1}H, δ 7.26), and $CDCl_3$ (for ^{13}C, δ 77.00). ^{1}H NMR data are reported as follows: chemical shift, integration, multiplicity (s = singlet, d = doublet, t = triplet, q = quartet, sext = sextet, sept = septet, dd = double doublet, dt = double triplet, dq = double quartet, ddt = double double triplet, br = broad, m = multiplet) and coupling constants (Hz). Infrared spectra were recorded on a JASCO FT/IR-4100 spectrometer. High-resolution mass spectra analysis was performed on a Bruker Daltonics solariX FT-ICR-MS spectrometer at the Research and Analytical Center for Giant Molecules, Graduate School of Science, Tohoku University. Flash column chromatography was performed with Kanto Chemical silica gel 60N (spherical, neutral, 40–50 μm). Analytical thin layer chromatography (TLC) was performed on Merck precoated TLC plates (silica gel 60 F_{254}). All reactions were carried out under argon atmosphere.

Preparative procedure for the starting material **1**, general procedures for the cobalt-catalyzed reaction of **1**, and spectroscopic data for starting materials **1a–d**, **1f**–**i, 1k**, **1m–v**, **1y–ab**, and products **2a–i**, **2k**, **2o–y'** are described in Supporting Information of our previous report [S1].

Materials

$CoCl_2$ was purchased from Wako and used as received.

$AgSbF_6$ was purchased from Aldrich and used as received.

Water-18 (98% ^{18}O) was purchased from ISOTEC and used as received.

2. Thermally induced reaction of 1y'

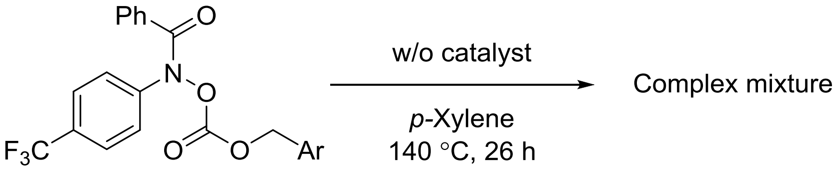

1y'
Ar = p-$O_2NC_6H_4$

3. Crossover experiment analyzed by HRMS [S1]

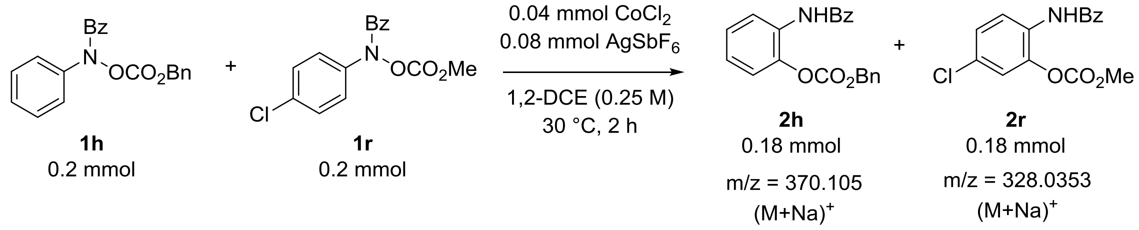

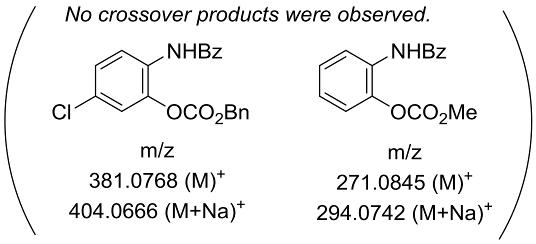

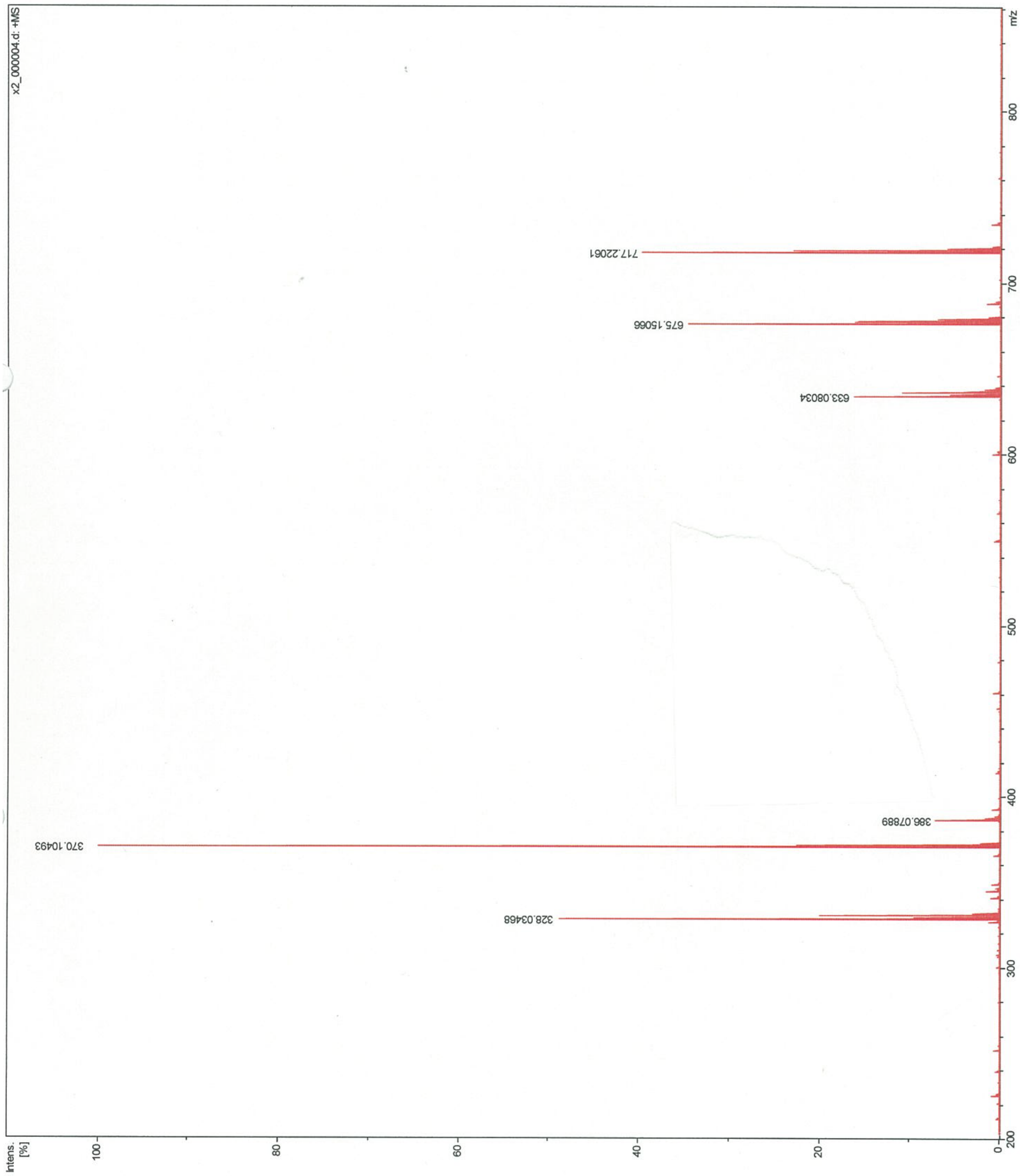

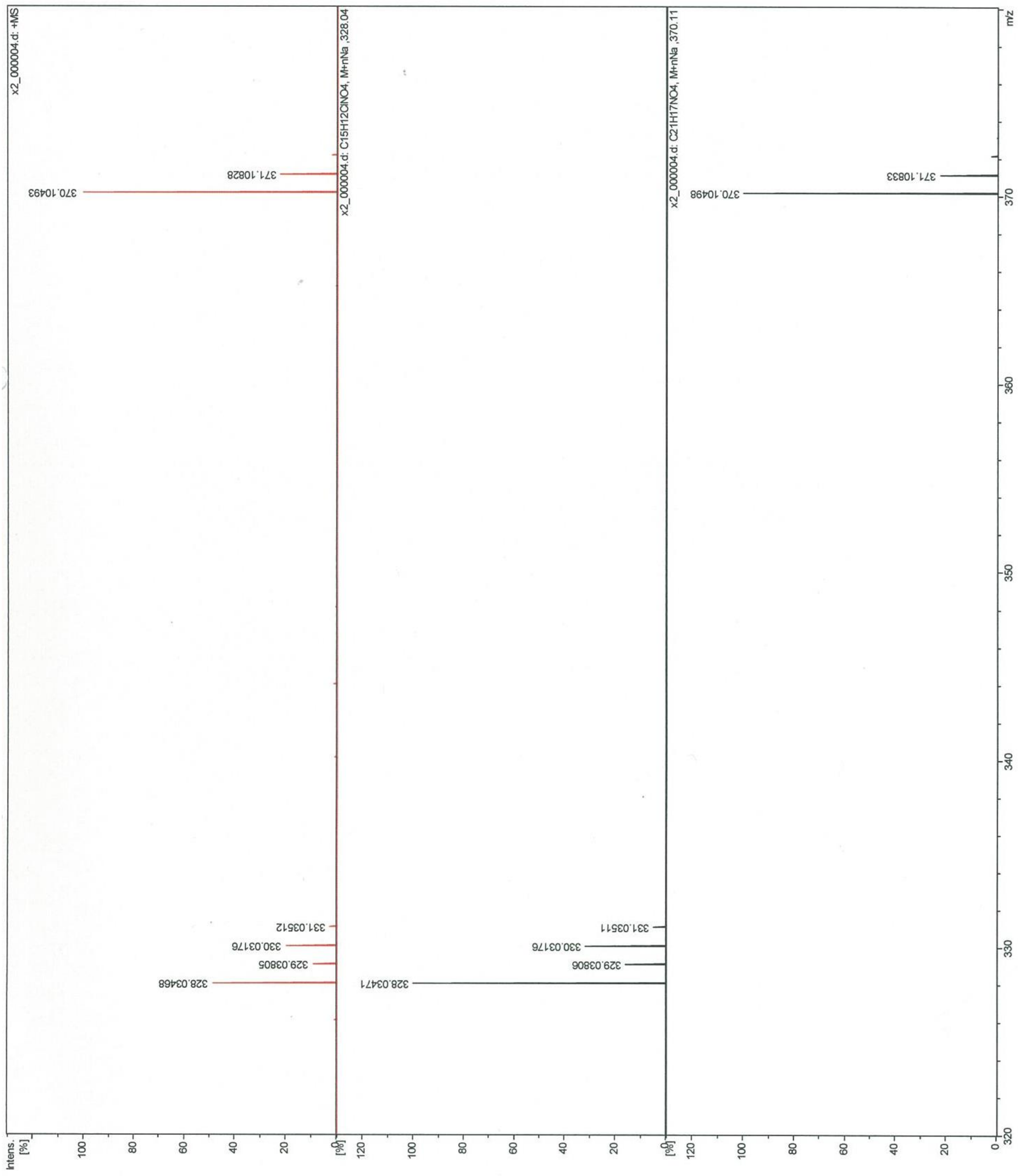

x2_000004.d: +MS
x2_000004.d: C15H12ClNO4, M+nNa ,328.04
x2_000004.d: C21H17NO4, M+nNa ,370.11
370.10493
371.10828
328.03468
329.03805
330.03176
331.03512
328.03471
329.03806
330.03176
331.03511
370.10498
371.10833
Intens. [%]
m/z

4. Intramolecular competitive experiments [S1]

Synthesis of nitrobenzene-d_1

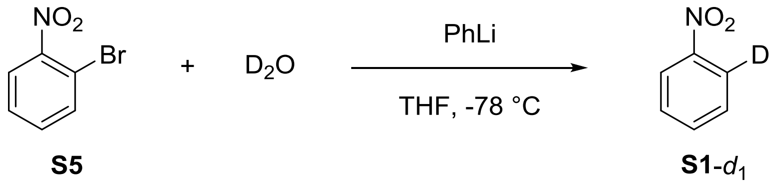

To a solution of **S5** (3.0 g, 15 mmol) in THF (50 mL) under argon atmosphere was added dropwise a 1.6 M butyl ether solution of phenyllithium (9.4 mL, 15 mmol) at −78 °C. After stirring the solution at -78 °C for 5 hours, the reaction was quenched by saturated aqueous sodium chloride. Then, the mixture was extracted with ether and the organic layer was washed by brine and dried over sodium sulfate. After the solvent was removed in vacuo, the residue was purified by distillation to obtain **S1**-d_1 (89% D, 0.9 mmol, 6% yield).

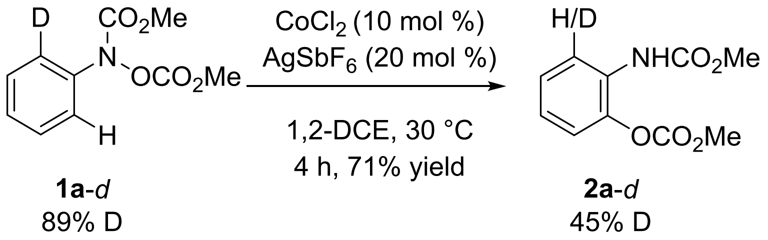

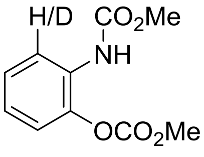

2a-*d*

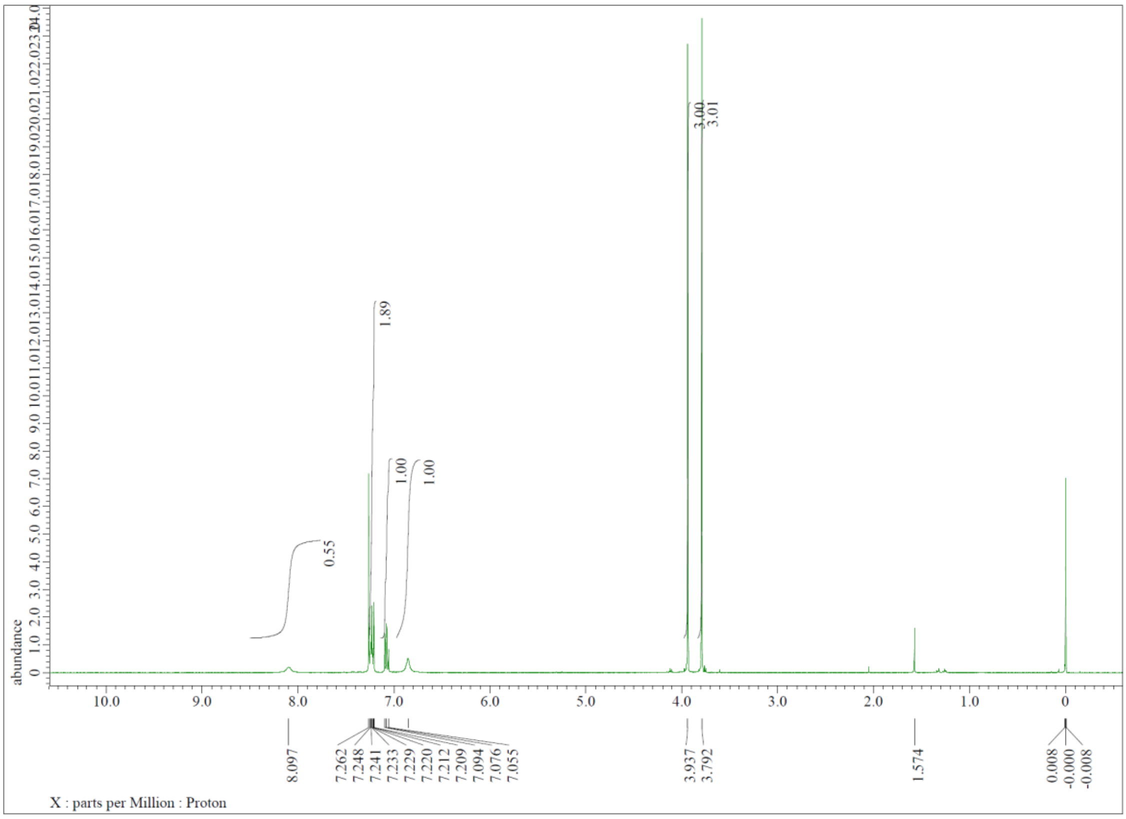

abundance
X : parts per Million : Proton
8.097
7.262
7.248
7.241
7.233
7.229
7.220
7.212
7.209
7.094
7.076
7.055
3.937
3.792
1.574
0.008
-0.000
-0.008
0.55
1.89
1.00
1.00
3.00
3.01

5. ^{18}O-Labeling experiments [S1]

a) Preparation of nitrobenzene-^{18}O

^{18}O-labelled sodium nitrate [S2] and nitrobenzene-^{18}O [S3] were prepared according to a reported procedure. ^{18}O content was determined by intensity of mass spectra.

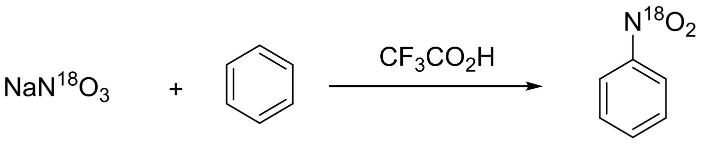

S1-^{18}O

To water-^{18}O was (0.3 mL, 16.6 mmol) in a 10 mL flask was added fumed nitric acid (d = 1.5, 0.1 mL, 2.4 mmol) and the solution in the tightly sealed flask was stirred at room temperature for 4 days. Then, to the solution was added sodium hydroxide (382 mg) in water-^{18}O (0.344 mL) and stirred for 24 hours. After water-^{18}O was distilled, fumed nitric acid (0.016 mL) was added to the residue. The mixture was stirring at 70 °C for 24 hours. After confirming that the solution was not acidic by pH test paper, water-^{18}O was distilled out. To the residue was added benzene (0.14 mL, 1.53 mmol) and trifluoroacetic acid (7.7 mL). The solution was stirred at room temperature for 24 hours. The reaction was quenched by 3 N NaOH in water-^{18}O until the solution was neutralized. The mixture was extracted three times with ether. Ether of the organic layer was distilled out. The obtained nitrobenzene-^{18}O (^{18}O 56%) was utilized without further purification.

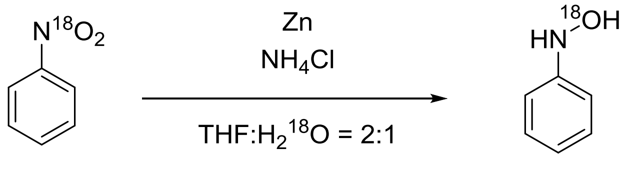

S1-^{18}O **S2**-^{18}O

To a solution of nitrobenzene-^{18}O in THF (3.6 mL) were added water-^{18}O (1.8 mL) and ammonium chloride (52.96 mg) at 0 °C under argon atmosphere. Then, the solution was added portion-wise zinc power (91.53 mg). After the solution was stirred at 0 °C for 2.5 hours, The reaction mixture was passed through a short pad of silica gel with ethyl acetate. After solvents were removed in vacuo, the crude product was purified by flash silica gel column chromatography using hexane/ethyl acetate (3/1) as eluents. After column chromatography, **S2**-^{18}O was not completely concentrated in order to avoid decomposition.

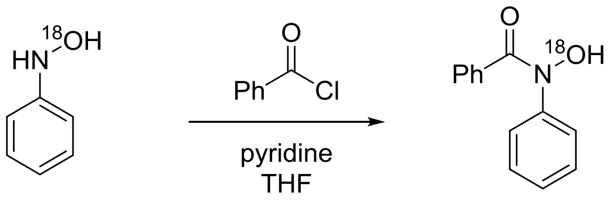

S2-^{18}O **S3**-^{18}O (72% ^{18}O)

To a solution of **S2**-^{18}O in THF (8 mL) was added pyridine at 0 °C under argon atmosphere. Benzoyl chloride was added dropwise to the solution. The solution was stirred at 0 °C with monitoring by TLC. After complete consumption of **S2**-^{18}O for 1 hour, the solution was quenched by ammonium chloride. The reaction mixture was passed through a short pad of silica gel with dehydrated dichloromethane. After removing solvents in vacuo, the crude product was purified by silica gel column chromatography using hexane/ethyl acetate (3/1 to 2/1) as eluent to obtain **S3**-^{18}O (72% ^{18}O, 0.4 mmol, 16% yield based on fumed nitric acid in the first step).

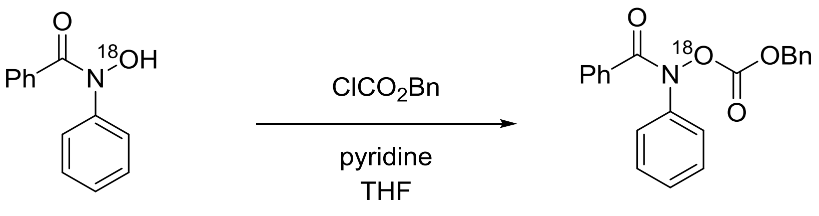

S3-^{18}O (72% ^{18}O) **1h**-^{18}O (62% ^{18}O)

To a solution of **S3**-^{18}O (94 mg, 0.396 mmol) in THF (8.8 mL) was added pyridine (36 μL, 0.44 mmol) at 0 °C under argon atmosphere. Then, benzyl chloroformate (64 μL, 0.45 mmol) was added dropwise to the solution. After 1 hour, the reaction was quenched by water and the reaction mixture was extracted with ether three times. The organic layer was washed with brine and dried over sodium sulfate. The crude product was purified by silica gel column chromatography to obtain **1h**-^{18}O (62% ^{18}O, 0.37 mmol, 84% yield).

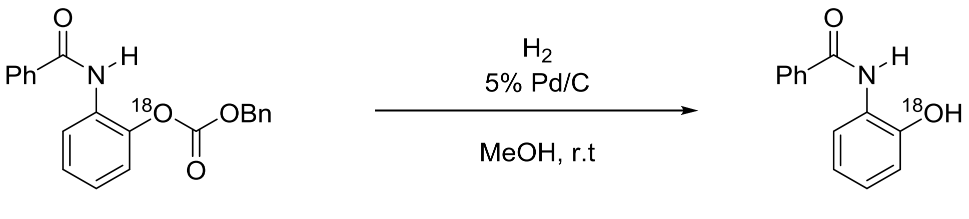

2h-^{18}O (62% ^{18}O) **4h**-^{18}O (64% ^{18}O)

To a solution of **2h**-^{18}O (34.8 mg, 0.10 mmol) in methanol (8.3 mL) in a 20 mL round-bottom flask was added 5% Pd/C under nitrogen atmosphere. Nitrogen gas was evacuated until

the solvent began to bubble, and then the flask was filled with hydrogen gas. The suspension was stirred at room temperature for 24 hours. The reaction mixture was filtered through celite and the filtrate was concentrated in vacuo. The residue was passed through a short pad of silica gel with ethyl acetate. The solution was washed with water and the water layer was extracted with ether and ethyl acetate. The combined organic layer was concentrated in vacuo to obtain **4h**-^{18}O (64% ^{18}O, 0.053 mmol, 53% yield) in an analytically pure form.

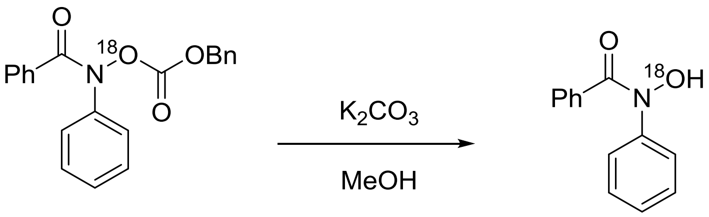

1h-^{18}O (62% ^{18}O)

S3-^{18}O (65% ^{18}O)

To **1h**-^{18}O (17.4 mg, 0.05 mmol) were added potassium carbonate (0.40 mg, 2.89 mmol) and methanol (0.3 mL). Then, the suspension was stirred for 6 hours. The mixture was purified by silica gel column chromatography using hexane/ethyl acetate (2.5:1) as eluent to obtain **S3**-^{18}O (65% ^{18}O) in 38% yield.

This result clearly indicates that oxygen-18 existed at the hydroxylamine moiety in **1h**-^{18}O.

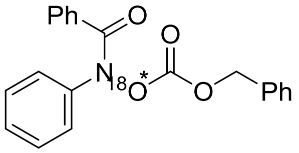

1h-^{18}O

m/z = 372.1092 $(M+Na)^+$

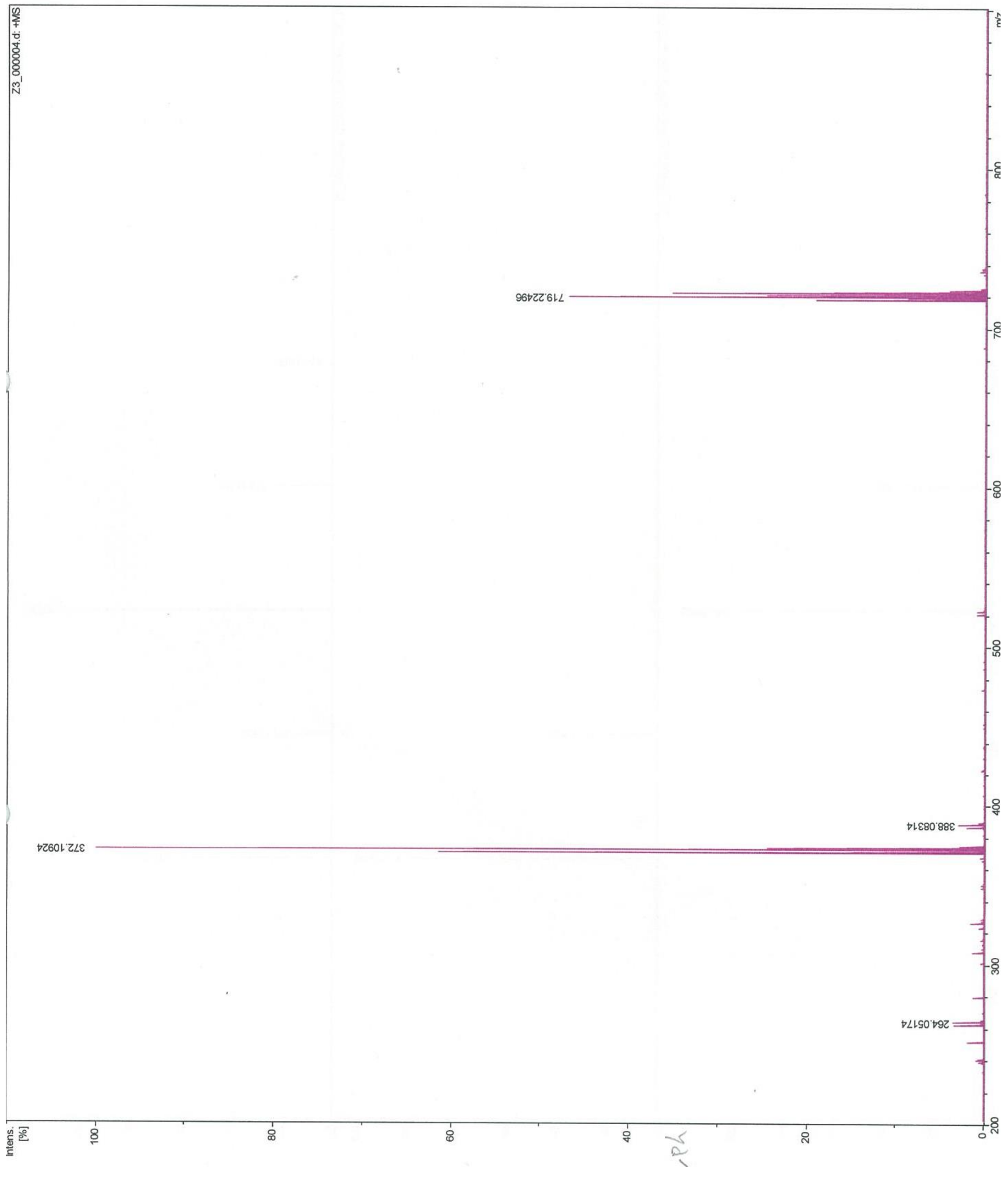

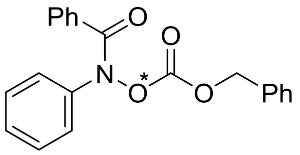

1h-^{18}O

m/z(calcd) = 372.1098 $(M+Na)^+$

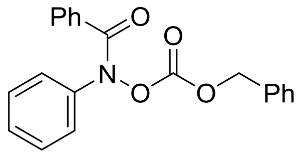

1h

m/z (calcd) = 370.1055 $(M+Na)^+$

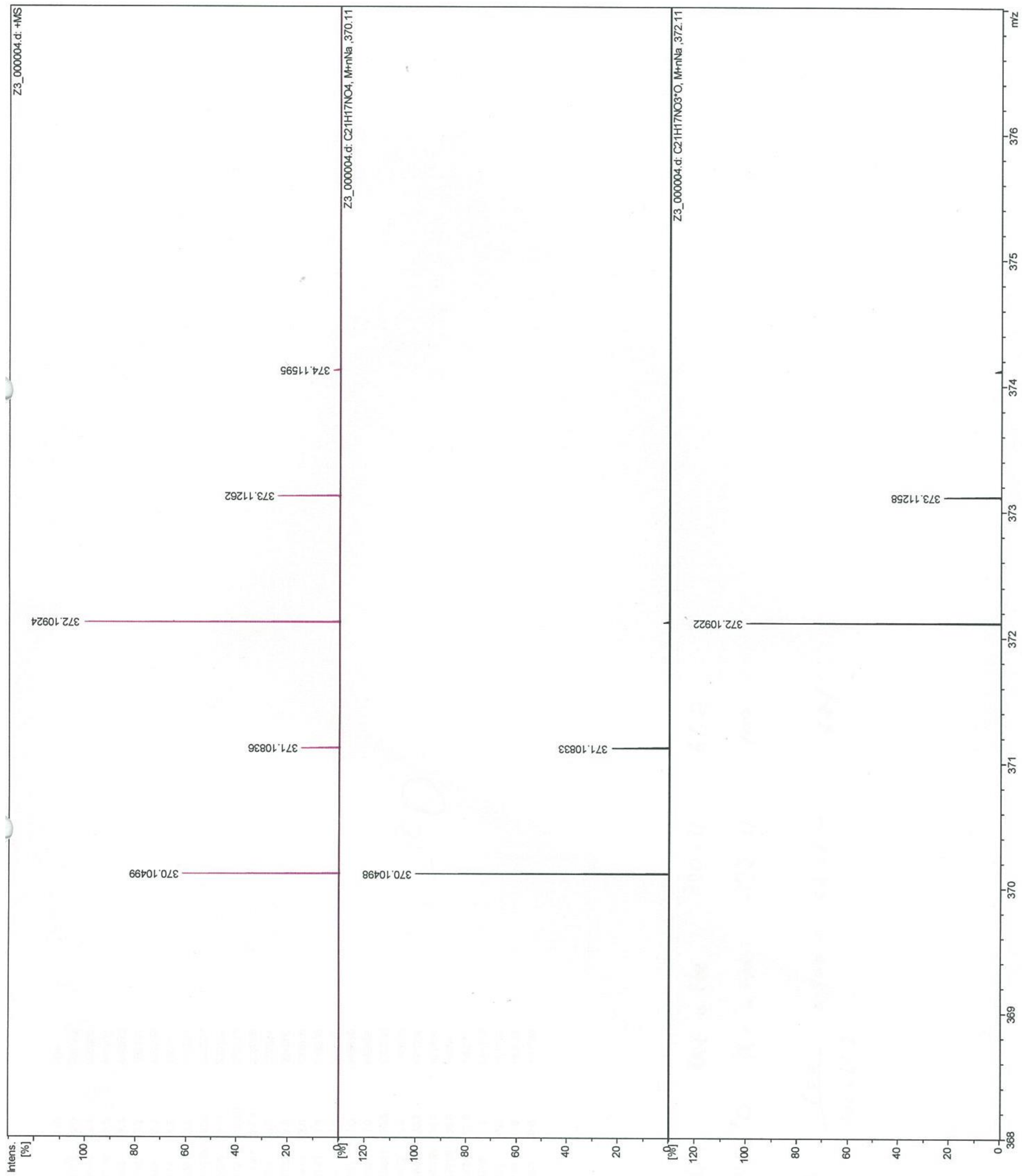

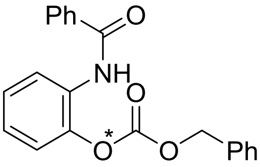

2h-^{18}O

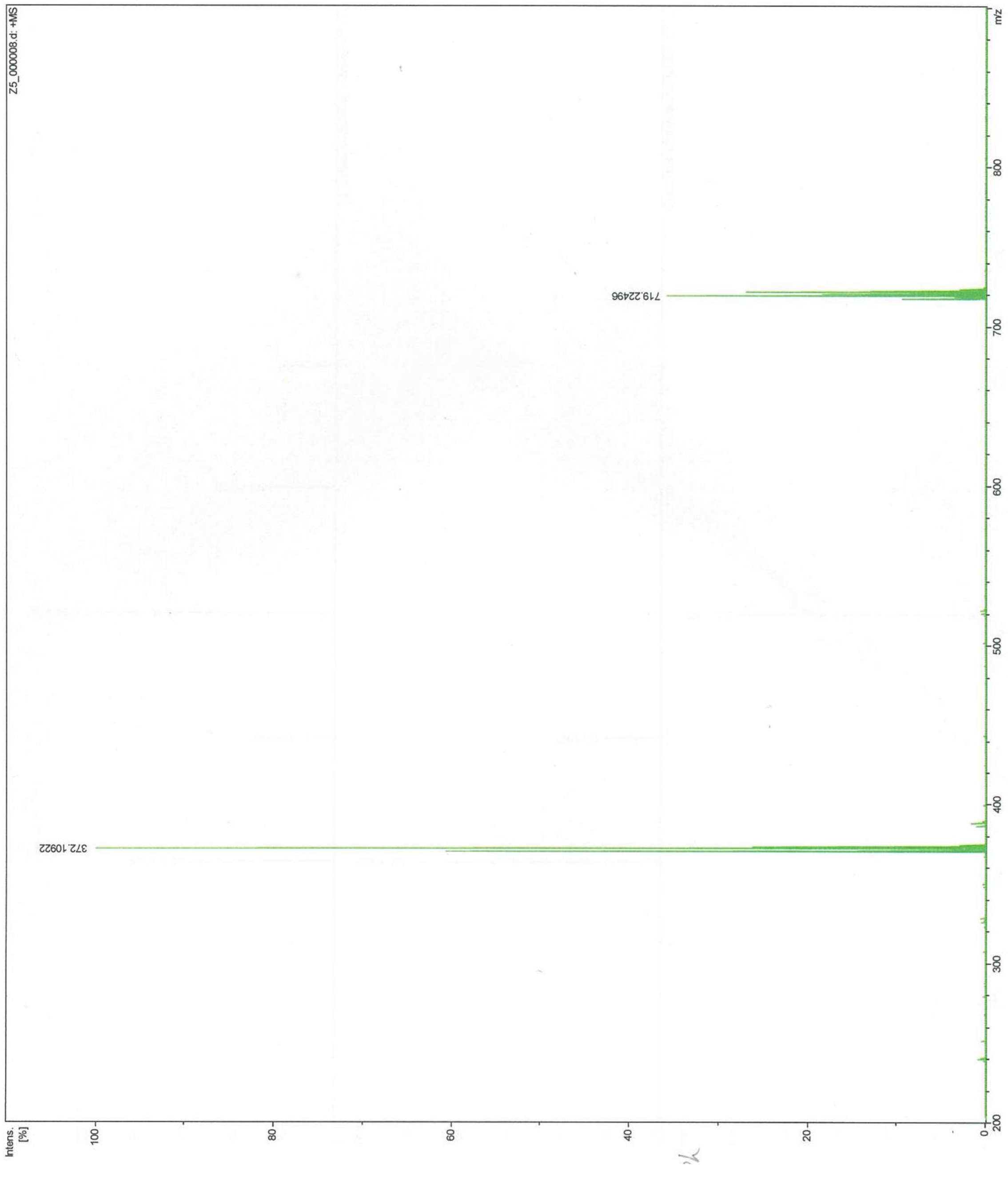

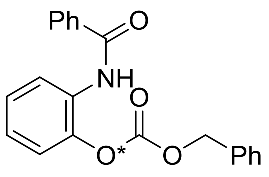

2h-^{18}O

m/z(calcd) = 372.1092 (M+Na)$^+$

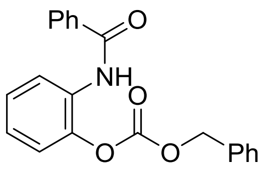

2h

m/z (calcd) = 370.1055 (M+Na)$^+$

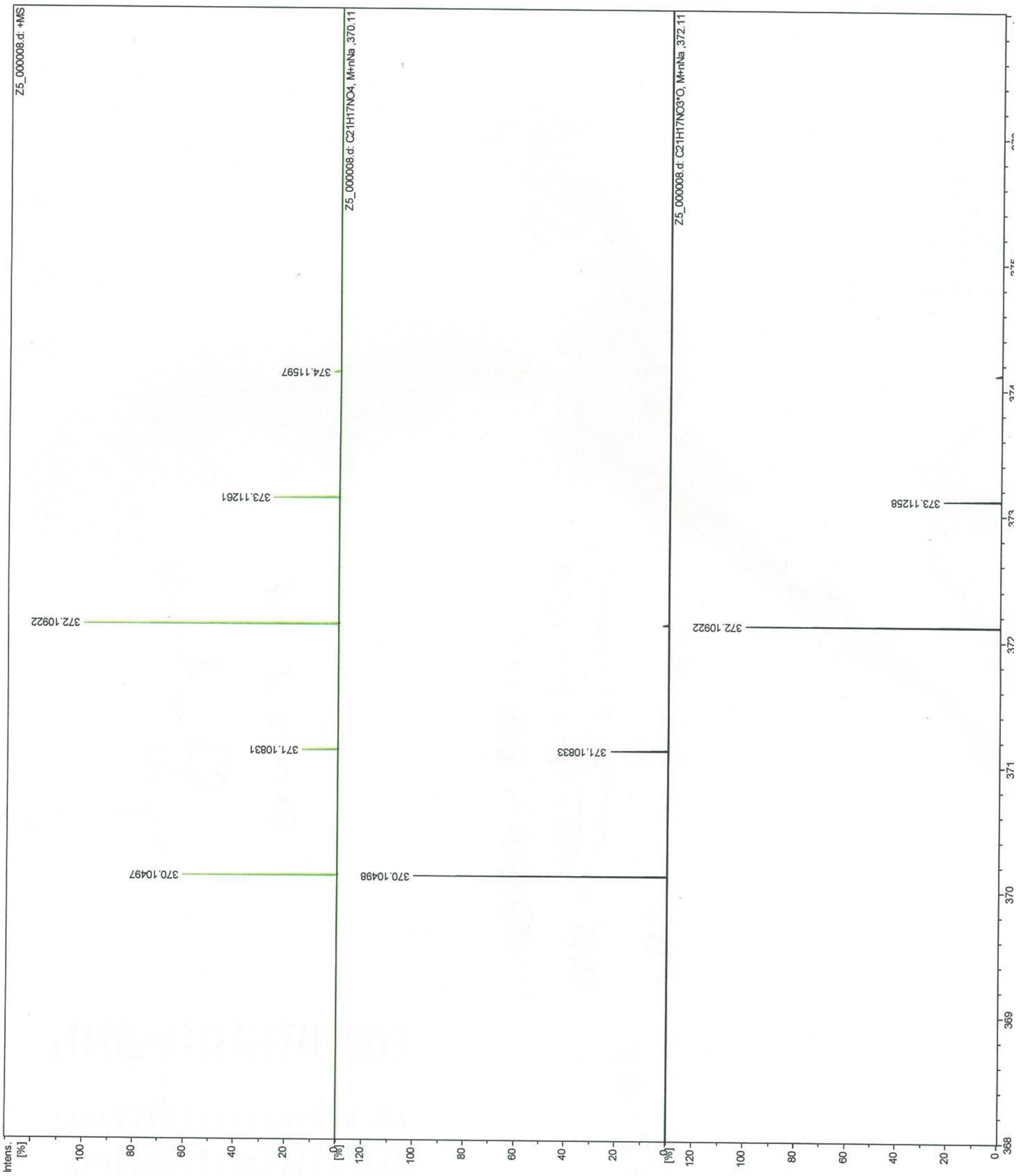

4h-^{18}O obtained by the Co-catalyzed reaction of **1h**-^{18}O followed by deprotection.

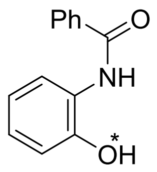

4h-^{18}O

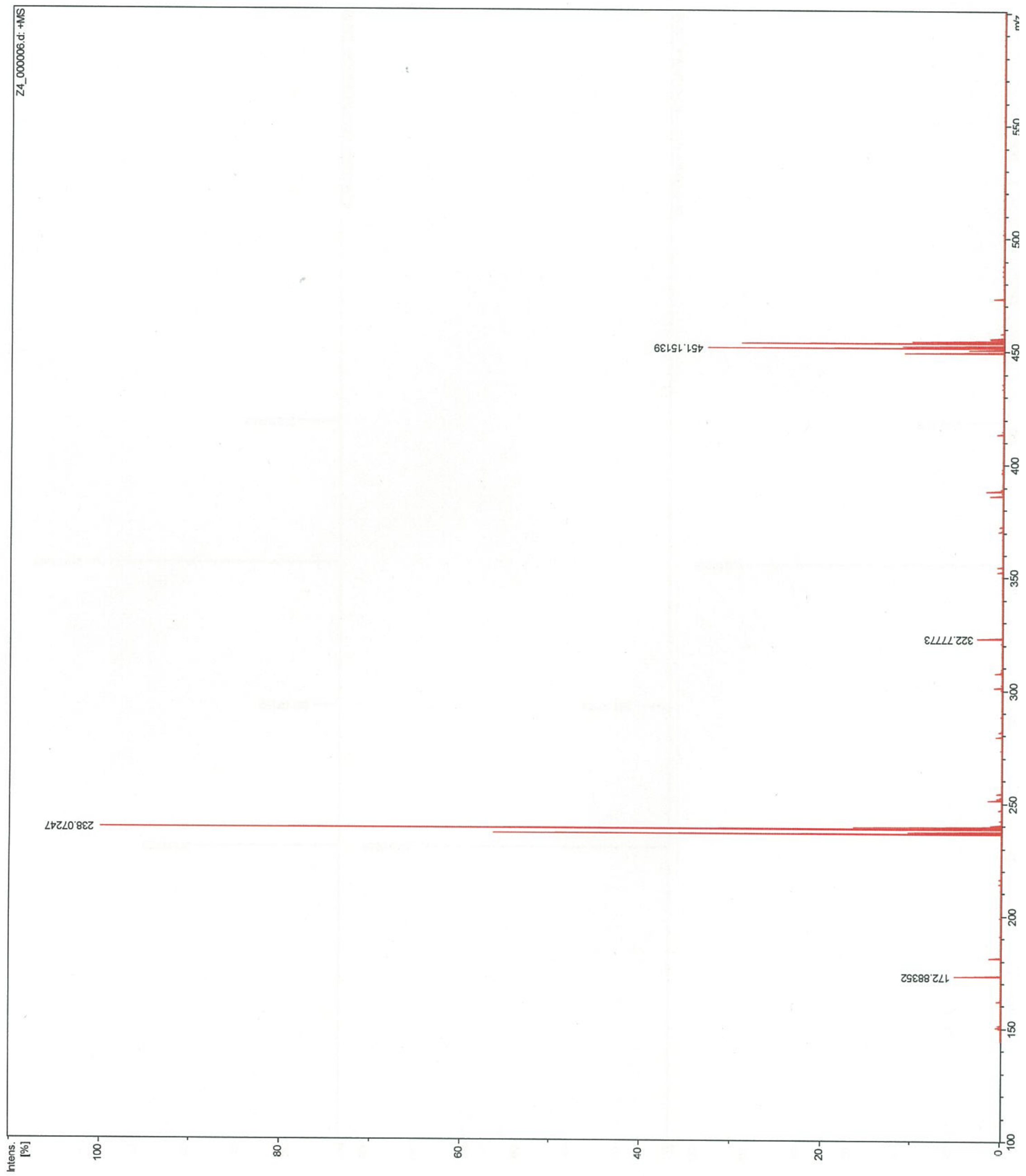

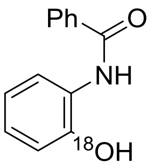

4h-^{18}O

m/z(calcd) = 238.0725 $(M+Na)^+$

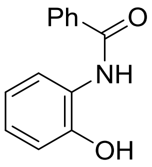

4h

m/z (calcd) = 236.0682 $(M+Na)^+$

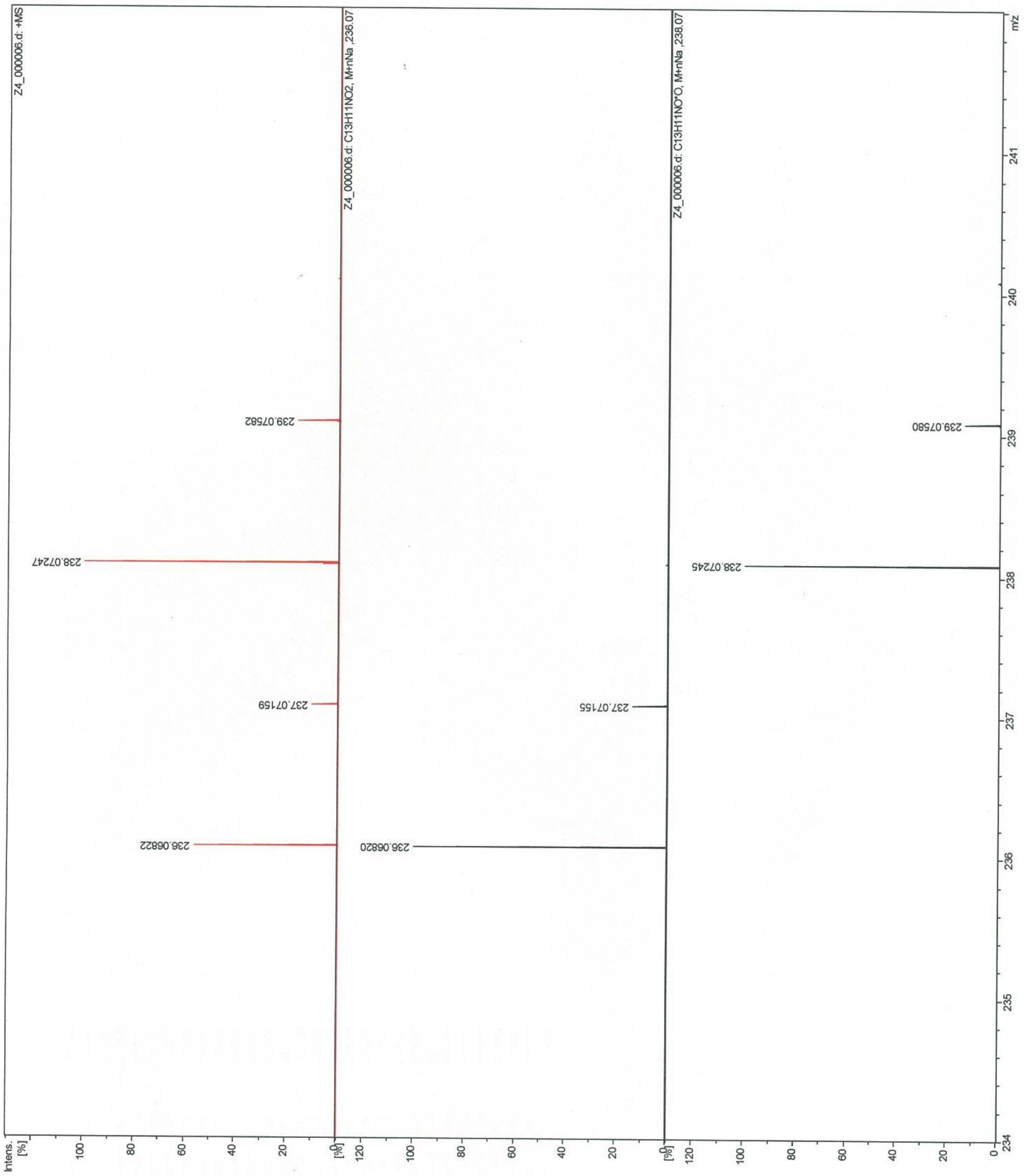

4h obtained by the thermal reaction of **1h**-^{18}O followed by deprotection

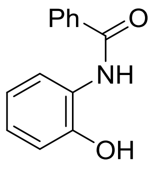

4h

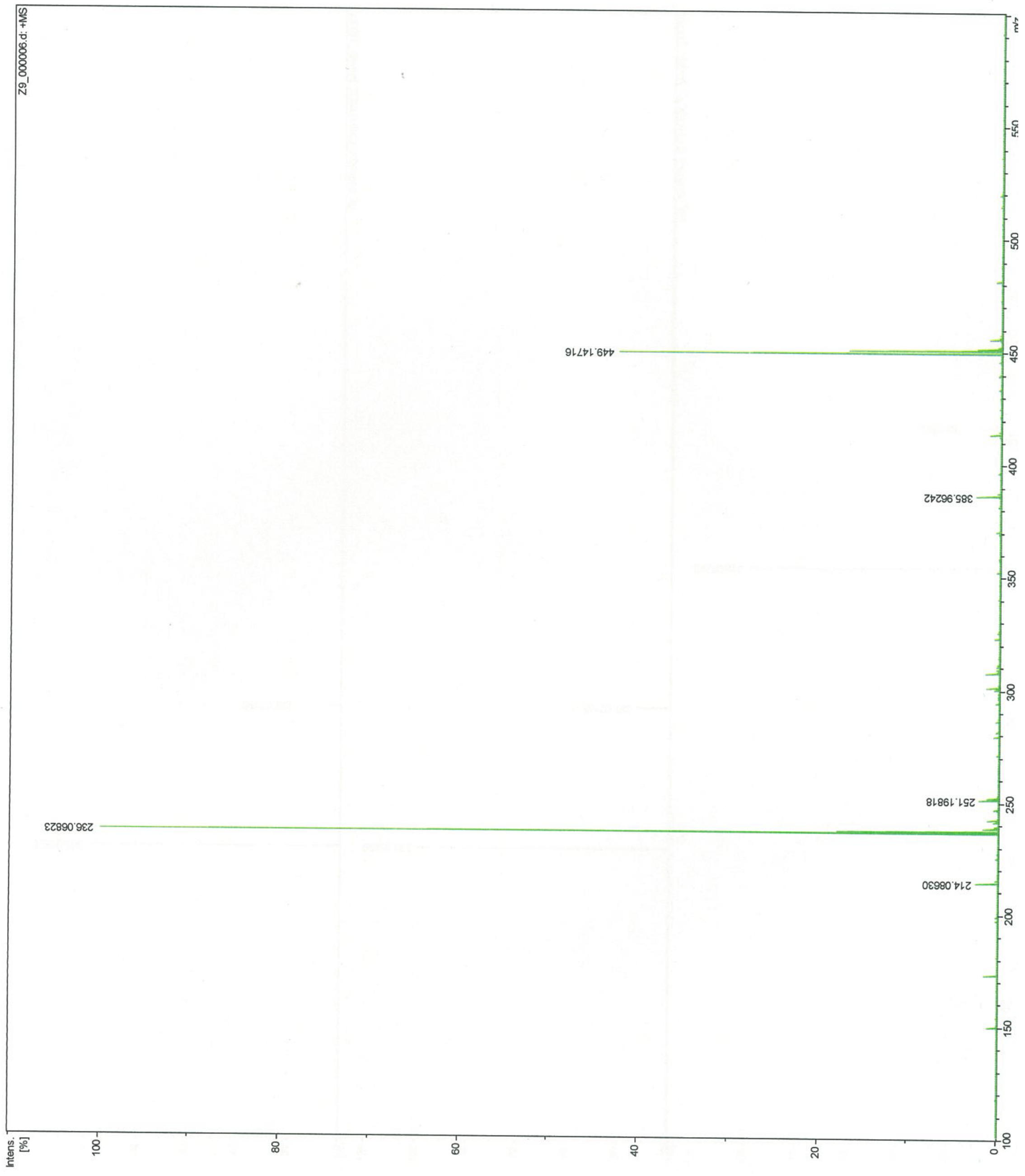

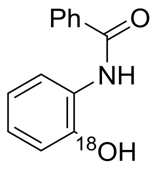

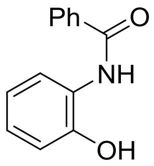

4h-^{18}O   **4h**

m/z(calcd) = 238.0725 $(M+Na)^+$ m/z (calcd) = 236.0682 $(M+Na)^+$

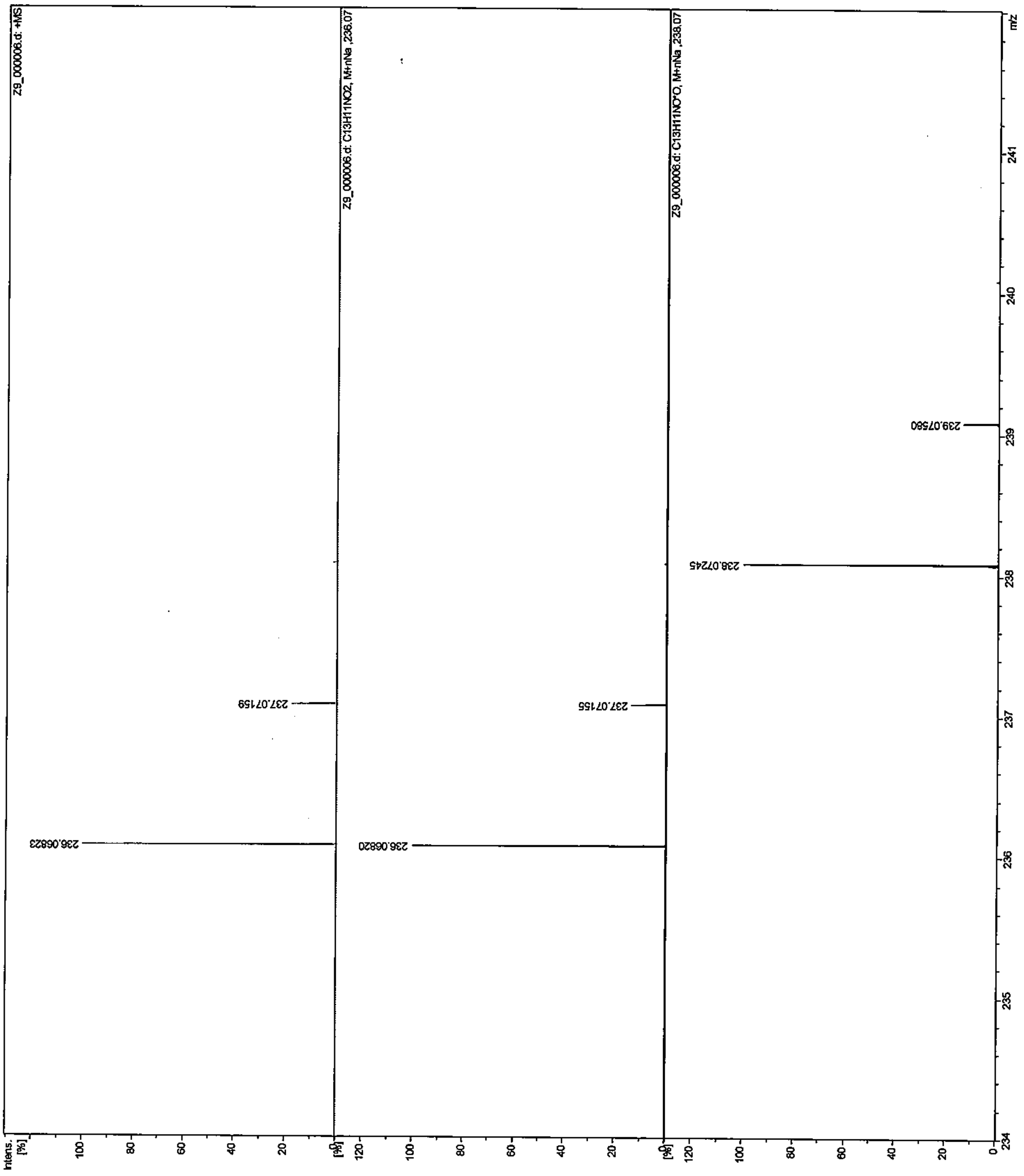

6. Analytical data of 1

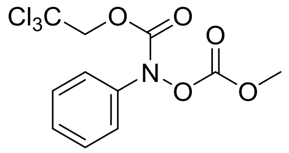

1e

***N*-phenyl-*N*-(2,2,2-trichloroethylcarboxyl)-*O*-(methoxycarbonyl)hydroxylamine (1e).** ^{1}H NMR (400 MHz, $CDCl_3$) δ 4.82 (s, 2H), 5.28 (s, 2H), 7.32 (t, J = 7.3 Hz, 1H), 7.37 (s, 5H), 7.41 (t, J = 7.3 Hz, 2H), 7.51 (d, J = 7.8 Hz, 2H). ^{13}C NMR (100 MHz, $CDCl_3$) δ 71.84, 75.54, 94.42, 124.29, 128.18, 128.55, 128.71, 129.06, 133.82, 138.42, 151.52, 153.79. IR (neat) 3067, 3035, 2958, 2893, 1794, 1739, 1596, 1493, 1456, 1385, 1341, 1308, 1216 , 1159, 1118, 1051, 1030, 954, 901, 827, 795, 772, 752, 735, 717, 694 cm^{-1}. HRMS (ESI) calcd. for $(M+Na)^+$ 439.9830, found. 439.9830.

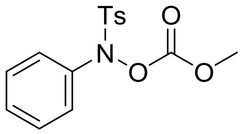

1j

***N*-Phenyl-*N*-(4-toluenesulfonyl)-*O*-(methoxycarbonyl)hydroxylamine (1j).** ^{1}H NMR (400 MHz, $CDCl_3$) δ 2.41 (s, 3H), 5.25 (s, 2H), 7.13 (m, 2H), 7.21 (d, J = 8.2 Hz, 2H), 7.25-7.29 (m, 3H), 7.36 (s, 5H), 7.45 (d, J = 8.2 Hz, 2H). ^{13}C NMR (100 MHz, $CDCl_3$) δ 21.69, 71.48, 124.00, 128.51, 128.59, 128.65, 128.89, 129.33, 129.43, 129.57, 129.74, 134.05, 139.85, 145.53, 153.03. IR (neat) 3067, 3035, 2959, 1596, 1487, 1455, 1089, 1029, 948, 910, 814, 795, 772, 757, 708, 658 cm^{-1}. HRMS (ESI) calcd. for $(M+Na)^+$ 420.0876, found. 420.0876.

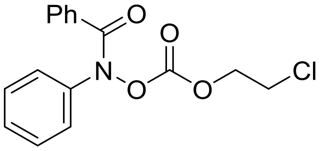

1l

***N*-Benzoyl-*N*-phenyl-*O*-(2-chloroethoxycarbonyl)hydroxylamine (1l).** ^{1}H NMR (400 MHz, $CDCl_3$) δ 3.70 (t, J = 5.9 Hz, 2H), 4.48 (t, J = 5.9 Hz, 2H), 7.25-7.34 (m, 7H), 7.38 (t, J = 7.5 Hz, 1H), 7.53 (d, J = 7.3 Hz, 2H). ^{13}C NMR (100 MHz, $CDCl_3$) δ 40.65, 68.81, 127.06, 128.17, 128.84, 128.88, 129.28, 131.38, 132.59, 139.97, 153.50, 166.94. IR (neat) 3063, 3027, 2963, 1788, 1678, 1593, 1489, 1448, 1385, 1336, 1306, 1219, 1141, 1075, 993, 915, 846, 768, 694, 661, 611 cm^{-1}. HRMS (ESI) calcd. for $(M+Na)^+$ 342.0503, found. 342.0503.

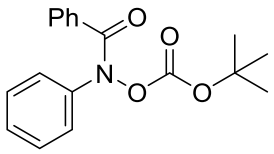

1m

***N*-Benzoyl-*N*-phenyl-*O*-(*tert*-butoxycarbonyl)hydroxylamine (1m).** ^{1}H NMR (400 MHz, $CDCl_3$) δ 1.48 (s, 9H), 7.28-7.41 (m, 8H), 7.57 (d, J = 7.3 Hz, 2H). ^{13}C NMR (100 MHz, $CDCl_3$) δ 27.48, 85.89, 125.99, 128.05, 128.14, 128.67, 129.09, 131.03, 133.29, 140.14, 151.62, 167.30. IR (neat) 3453, 3058, 2981, 2932, 1785, 1681, 1592, 1578, 1489, 1452, 1369, 1342, 1296, 1269, 1245, 1141, 1050, 1015, 929, 850, 795, 700 cm^{-1}. HRMS (ESI) calcd. for $(M+Na)^+$ 336.1206, found. 336.1206.

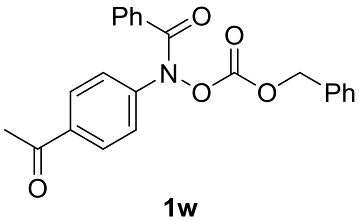

1w

***N*-Benzoyl-*N*-(4-acetylphenyl)-*O*-(benzyloxycarbonyl)hydroxylamine (1w).**^{1}H NMR (400 MHz, $CDCl_3$) δ 2.58 (s, 3H), 5.24 (s, 2H), 7.28-7.38 (m, 7H), 7.42-7.46 (m, 3H), 7.55-7.57 (m, 2H), 7.92 (d, J = 9.2 Hz, 2H). ^{13}C NMR (100 MHz, $CDCl_3$) δ 26.59, 71.86, 123.92, 128.32, 128.36, 128.56, 128.69, 129.00, 129.25, 131.67, 132.62, 133.71, 135.53, 143.70, 153.64, 167.23, 196.74.IR (neat) 3350, 3065, 3031, 3011, 2965, 2921, 1790, 1682, 1599, 1504, 1448, 1416, 1358, 1326, 1297, 1266, 1218, 1147, 1054, 958, 899, 836, 768, 697, 658 cm^{-1}. HRMS (ESI) calcd. for $(M+Na)^+$ 412.1155, found. 412.1155.

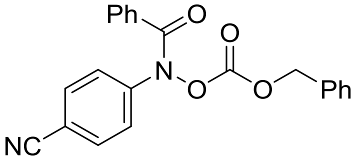

1x

***N*-Benzoyl-*N*-(4-cyanophenyl)-*O*-(benzyloxycarbonyl)hydroxylamine (1x).**^{1}H NMR (400 MHz, $CDCl_3$) δ 5.21 (s, 2H), 7.24-7.26 (m, 2H), 7.32-7.40 (m, 5H), 7.45-7.57 (m, 5H), 7.64 (d, J = 8.7 Hz, 2H). ^{13}C NMR (150 MHz, $CDCl_3$) δ 72.08, 110.43, 118.02, 123.26, 128.37(br s), 128.39, 128.69, 129.08, 131.84, 132.36, 132.96, 133.52, 143.38, 153.47, 167.51. IR (neat) 3900, 3845, 3794, 3666, 3416, 3213, 3202, 3066, 3042, 2229, 1793, 1687, 1602, 1502, 1447, 1414, 1329, 1301, 1263, 1220, 1142, 1050, 898, 833, 696 cm^{-1}. HRMS (ESI) calcd. for $(M+Na)^+$ 395.1002, found. 395.1002.

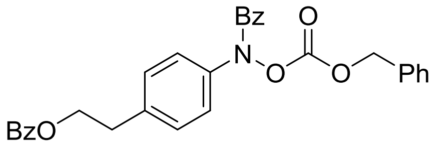

1z

4-(N-(((Benzyloxy)carbonyl)oxy)benzamido)phenethyl benzoate (1z). ^{1}H NMR (400 MHz, $CDCl_3$) δ 3.05 (t, J = 6.9 Hz, 2H), 4.50 (t, J = 6.9 Hz, 2H), 5.25 (s, 2H), 7.59-7.21 (m, 17H), 7.97 (d, J = 7.3 Hz, 2H). ^{13}C NMR (100 MHz, $CDCl_3$) δ 34.79, 64.88, 71.51, 127.01, 128.12, 128.34, 128.40, 128.68, 128.77, 128.86, 129.53, 129.62, 130.05, 131.24, 132.89, 133.04, 134.08, 138.64, 138.85, 153.86, 166.41, 167.16. IR (neat) 3034, 2957, 2896, 2359, 2341, 1788, 1716, 1682, 1602, 1509, 1450, 1379, 1336, 1314, 1178, 1115, 1070, 1026, 968, 901, 843, 789, 739, 713, 698, 615 cm^{-1}. HRMS (ESI) calcd. for $(M+Na)^+$ 518.1574, found. 518.1574.

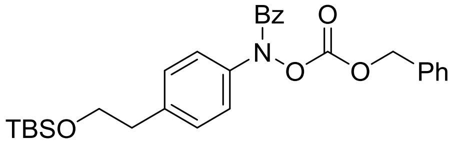

1aa

***N*-Benzoyl-*N*-[4-{2-(*tert*-butyldimethylsilyloxy)ethyl}phenyl]-*O*-(benzyloxycarbonyl)hydroxylamine (1aa).** ^{1}H NMR (400 MHz, $CDCl_3$) δ -0.08 (s, 6H), 0.83 (s, 9H), 2.77 (t, 2H), 3.76 (t, 2H), 5.26 (s, 2H), 7.15 (d, 2H), 7.24-7.27 (m, 6H), 7.35-7.38 (m, 6H), 7.53 (d, 2H). ^{13}C NMR (100 MHz, $CDCl_3$) -5.49, 18.22, 25.83, 39.00, 63.81, 71.36, 126.88, 128.09, 128.28, 128.62, 128.78, 130.00, 131.12, 132.99, 134.12, 138.20, 140.49, 153.81, 167.12. δ IR (neat) 3416, 3370, 3064, 3033, 2952, 2928, 2886, 2856, 1788, 1682, 1602, 1579, 1508, 1471, 1462, 1448, 1379, 1334, 1253, 1215, 1179, 1094, 1040, 1004, 966, 916, 901, 832, 774, 736, 695 cm^{-1}. HRMS (ESI) calcd. for $(M+Na)^+$ 528.2177, found. 528.2177.

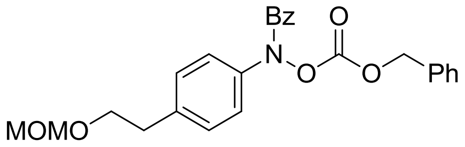

1ab

***N*-Benzoyl-*N*-[4-{2-(methoxymethoxy)ethyl}phenyl]-*O*-(benzyloxycarbonyl)hydroxylamine (1ab). (a mixture with 2ab (8.3 : 1)** ^{1}H NMR (400 MHz, $CDCl_3$) δ 2.87 (t, J = 6.9 Hz, 2H), 3.22 (s, 3H), 3.72 (t, J = 6.9 Hz, 2H), 4.57 (s, 2H), 5.25 (s, 2H), 7.18-7.20 (m, 2H), 7.23-7.28 (m, 4H), 7.32-7.38 (m, 6H), 7.52-7.55 (m, 2H). ^{13}C NMR (100 MHz, $CDCl_3$) δ 35.83, 55.17, 67.80, 71.44, 96.30, 127.02, 128.10, 128.33, 128.56, 128.66, 128.78, 128.84, 129.72, 131.17, 133.00, 134.10, 138.29, 140.11, 153.85. IR (neat) 3062, 3034, 2931, 2883, 2823, 1787, 1681, 1509, 1448, 1379, 1335, 1218, 1147, 1108, 1068, 1030, 965, 915, 845, 795, 770, 740, 698 cm^{-1}. HRMS (ESI) calcd. for $(M+Na)^+$ 458.1574, found. 458.1574.

7. Analytical data of 2

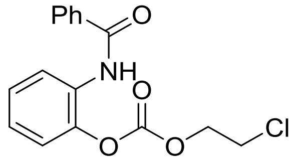

2l

2-[(Benzoyl)amino]phenyl 2-chloroethyl carbonate (2l). ^{1}H NMR (400 MHz, $CDCl_3$) δ 3.79 (t, J = 5.5 Hz, 2H), 4.55 (t, J = 5.5 Hz, 2H), 7.18 (t, J = 7.8 Hz, 1H), 7.31-7.34 (m, 2H), 7.51 (t, J = 7.3 Hz, 2H), 7.58 (t, J = 7.3 Hz, 1H), 7.89 (d, J = 7.3 Hz, 2H), 8.12 (br s, 1H), 8.42 (d, J = 8.2 Hz, 1H). ^{13}C NMR (100 MHz, $CDCl_3$) δ 41.17, 68.25, 121.40, 122.32, 124.60, 126.90, 127.08, 128.84, 129.73, 132.10, 134.37, 140.62, 152.48, 165.31. IR (neat) 3317, 3065, 2959, 1767, 1667, 1608, 1580, 1523, 1496, 1454, 1386, 1309, 1235, 1190, 1125, 1082, 1033, 998, 766, 740, 709 cm^{-1}. HRMS (ESI) calcd. for $(M+Na)^+$ 342.0503, found. 342.0503.

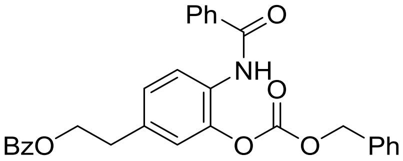

2z

4-Benzamido-3-(((benzyloxy)carbonyl)oxy)phenethyl benzoate (2z). Included with inseparable byproducts ^{1}H NMR (400 MHz, $CDCl_3$) δ 3.07 (t, J = 6.9 Hz, 2H), 4.52 (t, J = 6.9 Hz, 2H), 5.28 (s, 2H), 7.20-8.03 (m, 17H), 8.32 (d, J = 8.7 Hz, 1H). Reaction mixture was not purified by flash chromatography (Silica gel, hexanes:EtOAc = 3:1). Isolation of **2z** was unsuccessful due to contaimination by inseparable byproducts; further purification by using gel permeation chromatography resulted in decomposition of the product.

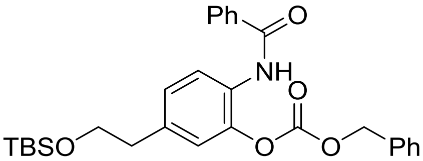

2aa

2-[(Benzoyl)amino]-4-[2-(*tert*-butyldimethylsiloxy)ethyl]phenyl 2-benzyl carbonate (2aa). ^{1}H NMR (400 MHz, $CDCl_3$) -0.01 (s, 6H), 0.90 (s, 9H), 2.83 (t, 2H), 3.82 (t, 2H), 5.29 (s, 2H), 7.15 (d, 2H), 7.35-7.37 (m, 3H), 7.42-7.45 (m, 4H), 7.78 (d, 2H), 8.04 (s, 1H), 8.26 (d, 1H). ^{13}C NMR (100 MHz, $CDCl_3$) -5.45, 18.28, 25.89, 38.86, 64.02, 70.82, 122.02, 122.34, 127.00, 127.45, 127.79, 128.51, 128.73, 128.76, 128.94, 131.86, 134.43, 134.56, 136.43, 140.89, 152.84, 165.19. IR (neat) 3444, 3314, 3065, 3033, 2952, 2928, 2895, 2856, 2737,

1766, 1679, 1601, 1520, 1481, 1421, 1379, 1312, 1208, 1130, 1093, 1045, 1028, 1005, 930, 908, 831, 774, 695 cm^{-1}. HRMS (ESI) calcd. for $(M+Na)^+$ 528.2177, found. 528.2177.

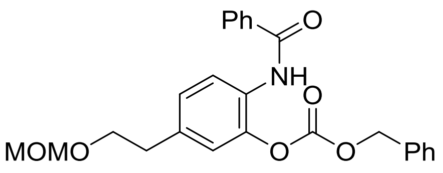

2ab

2-[(Benzoyl)amino]-4-[2-(methoxymethoxy)ethyl]phenyl 2-benzyl carbonate (2ab). ^1H NMR (400 MHz, $CDCl_3$) δ 2.90 (t, J = 6.9 Hz, 2H), 3.29 (s, 3H), 3.75 (t, J = 6.9 Hz, 2H), 4.63 (s, 2H), 5.28 (s, 2H), 7.17 (d, J = 9.6 Hz, 2H), 7.35-7.45 (m, 8H), 7.54 (t, J = 7.3 Hz, 1H), 7.77 (d, J = 7.3 Hz, 2H), 8.02 (br s, 1H), 8.26 (d, J = 8.2 Hz, 1H). ^{13}C NMR (100 MHz, $CDCl_3$) δ 35.59, 55.17, 67.94, 70.82, 96.31, 121.79, 122.40, 126.98, 127.15, 128.87, 128.51, 128.71, 128.75, 128.93, 131.34, 134.47, 136.03, 140.89, 152.80. IR (neat) 3323, 3062, 3033, 2934, 2884, 2824, 1766, 1675, 1602, 1581, 1521, 1483, 1421, 1380, 1311, 1234, 1212, 1147, 1108, 1070, 1029, 914, 795, 746, 697 cm^{-1}. HRMS (ESI) calcd. for $(M+Na)^+$ 458.1574, found. 458.1574.

8. Analytical data of 3

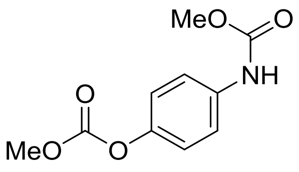

3a

4-[(Methoxycarbonyl)amino]phenyl methyl carbonate (3a) ^{1}H NMR (400 MHz, $CDCl_3$) δ 3.77 (s, 3H), 3.90 (s, 3H), 6.79 (br s, 1H), 7.09-7.12 (m, 2H), 7.39 (d, J = 8.2 Hz, 2H). ^{13}C NMR (100 MHz, $CDCl_3$) δ 52.40, 55.41, 119.38, 121.53, 135.73, 146.59, 153.94, 154.43. IR (neat) 3628, 3540, 3367, 3348, 3134, 3011, 2956, 2851, 1764, 1734, 1713, 1610, 1540, 1512, 1440, 1411, 1265, 1209, 1113, 1071, 1016, 948, 930, 839, 806, 771 cm^{-1}. HRMS (ESI) calcd. for $(M+Na)^+$ 248.0529, found. 248.0529.

9. ^{1}H and ^{13}C NMR chart of 1, 2, 3 and 4

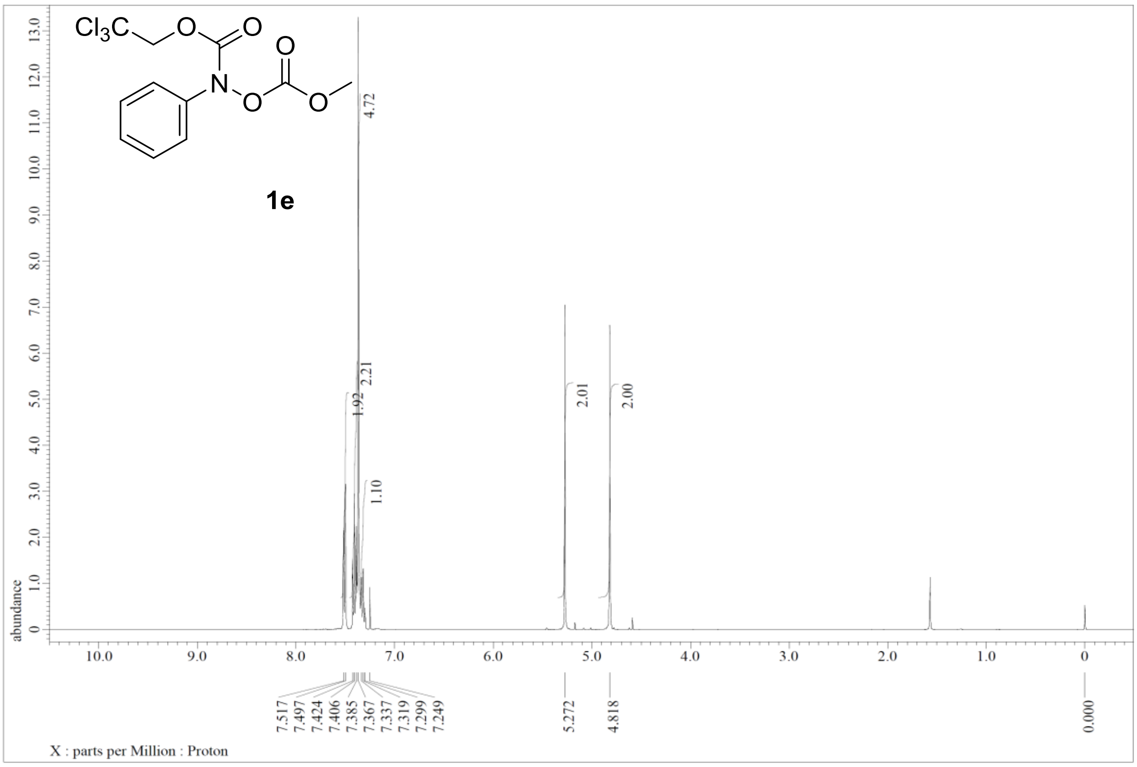

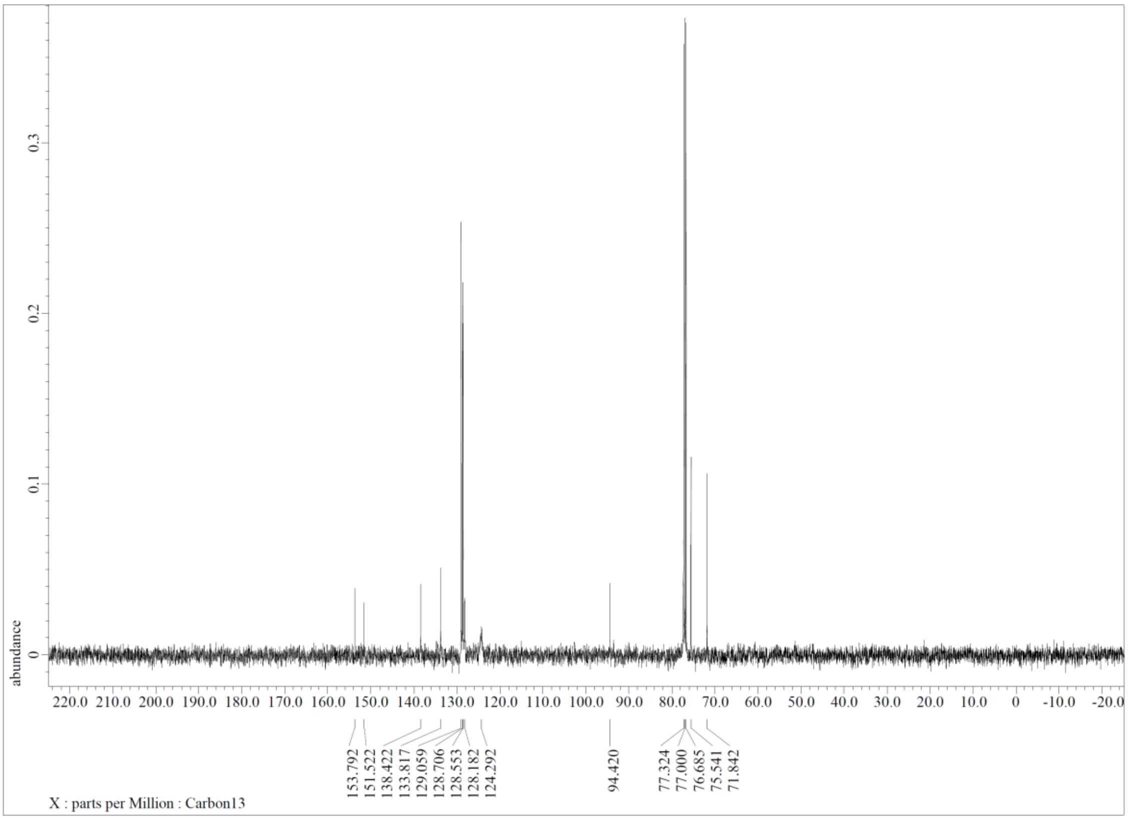

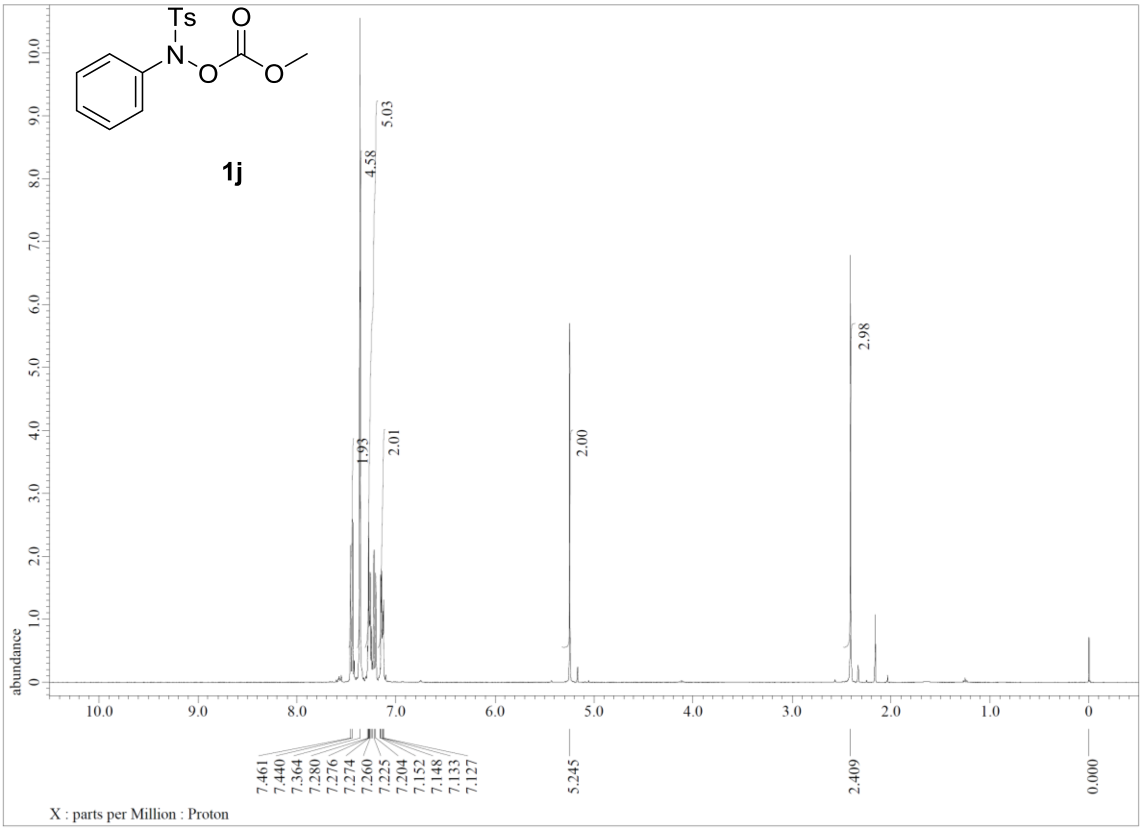

Ts
N
O
O
O
1j
abundance
1.93
4.58
5.03
2.01
2.00
2.98
7.461
7.440
7.364
7.280
7.276
7.274
7.260
7.225
7.204
7.152
7.148
7.133
7.127
5.245
2.409
0.000
X : parts per Million : Proton

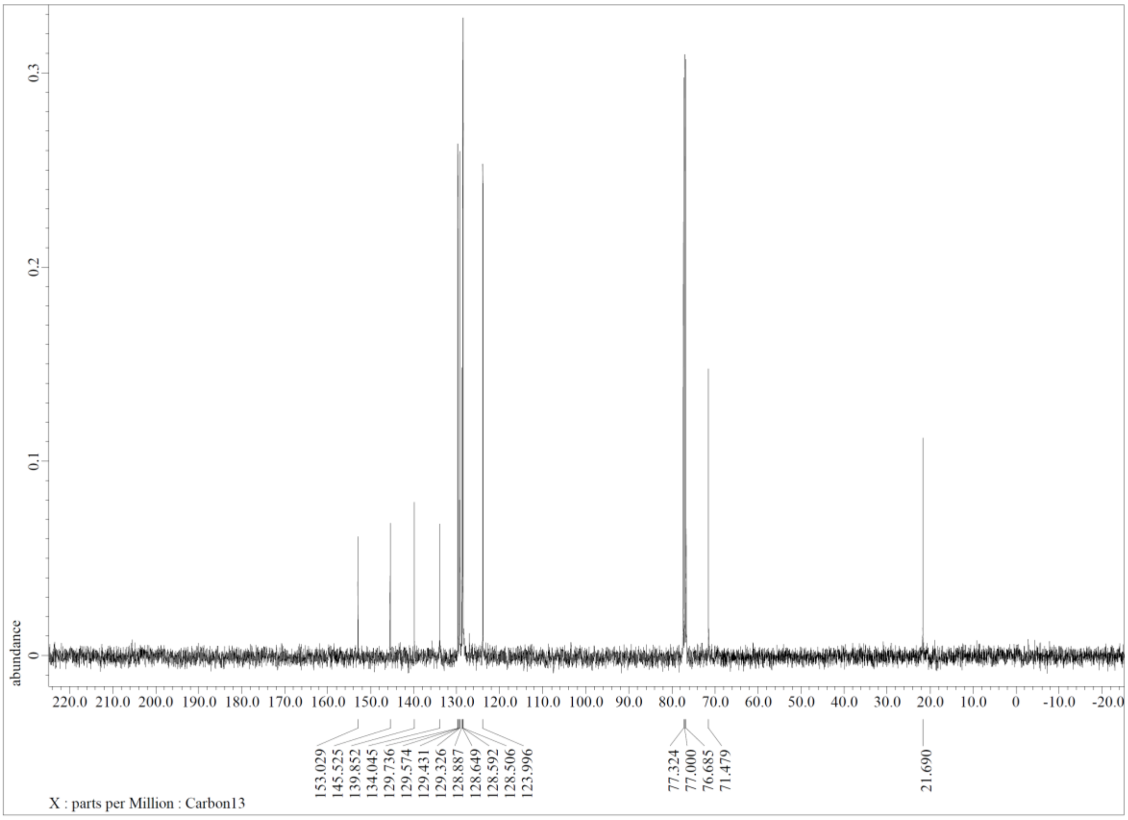

abundance
153.029
145.525
139.852
134.045
129.736
129.574
129.431
129.326
128.887
128.649
128.592
128.506
123.996
77.324
77.000
76.685
71.479
21.690
X : parts per Million : Carbon13

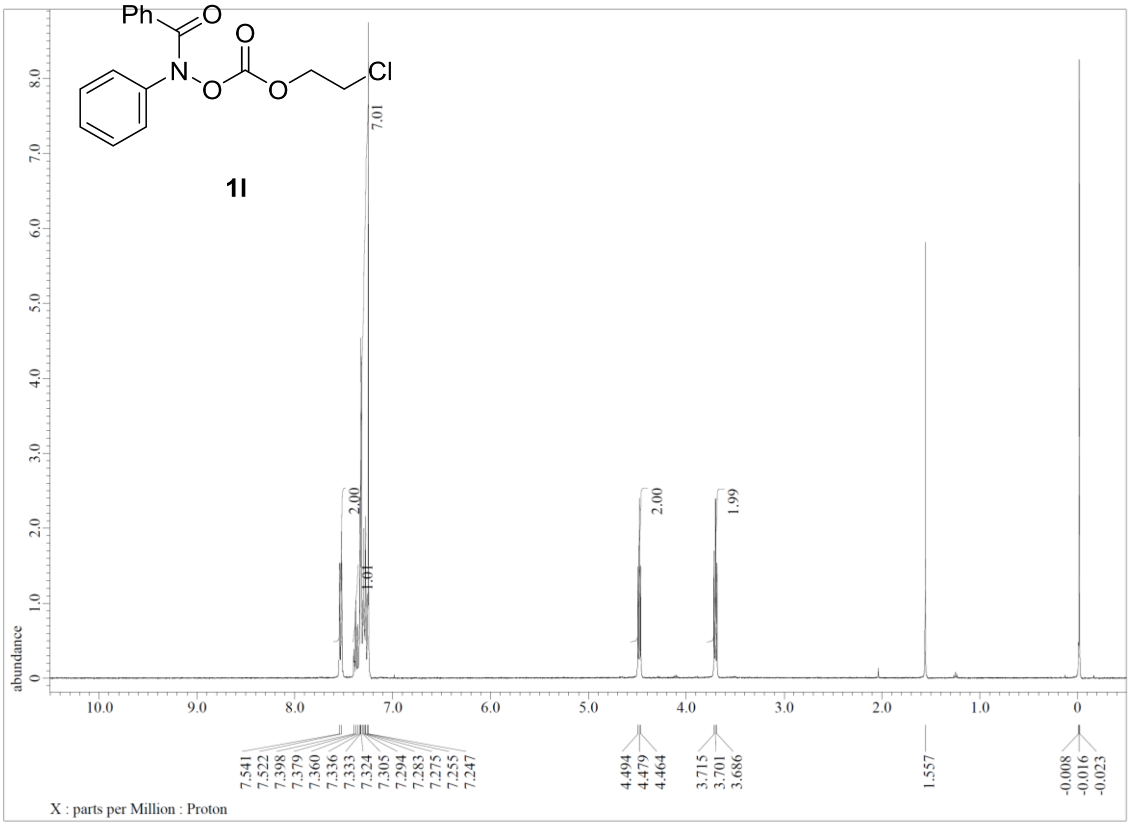
Ph
O
N
O
O
O
Cl
1l
7.01
2.00
1.01
2.00
1.99
abundance
X : parts per Million : Proton
7.541
7.522
7.398
7.379
7.360
7.336
7.333
7.324
7.305
7.294
7.283
7.275
7.255
7.247
4.494
4.479
4.464
3.715
3.701
3.686
1.557
-0.008
-0.016
-0.023

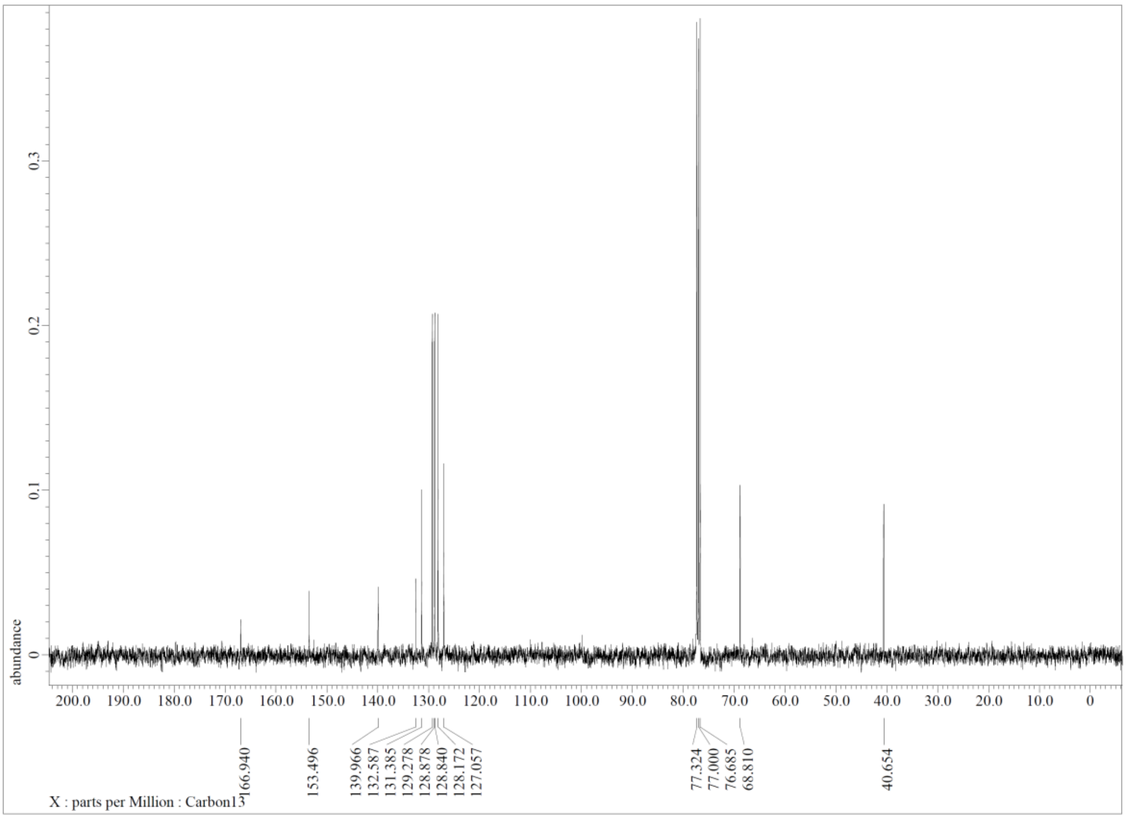
abundance
X : parts per Million : Carbon13
166.940
153.496
139.966
132.587
131.385
129.278
128.878
128.840
128.172
127.057
77.324
77.000
76.685
68.810
40.654

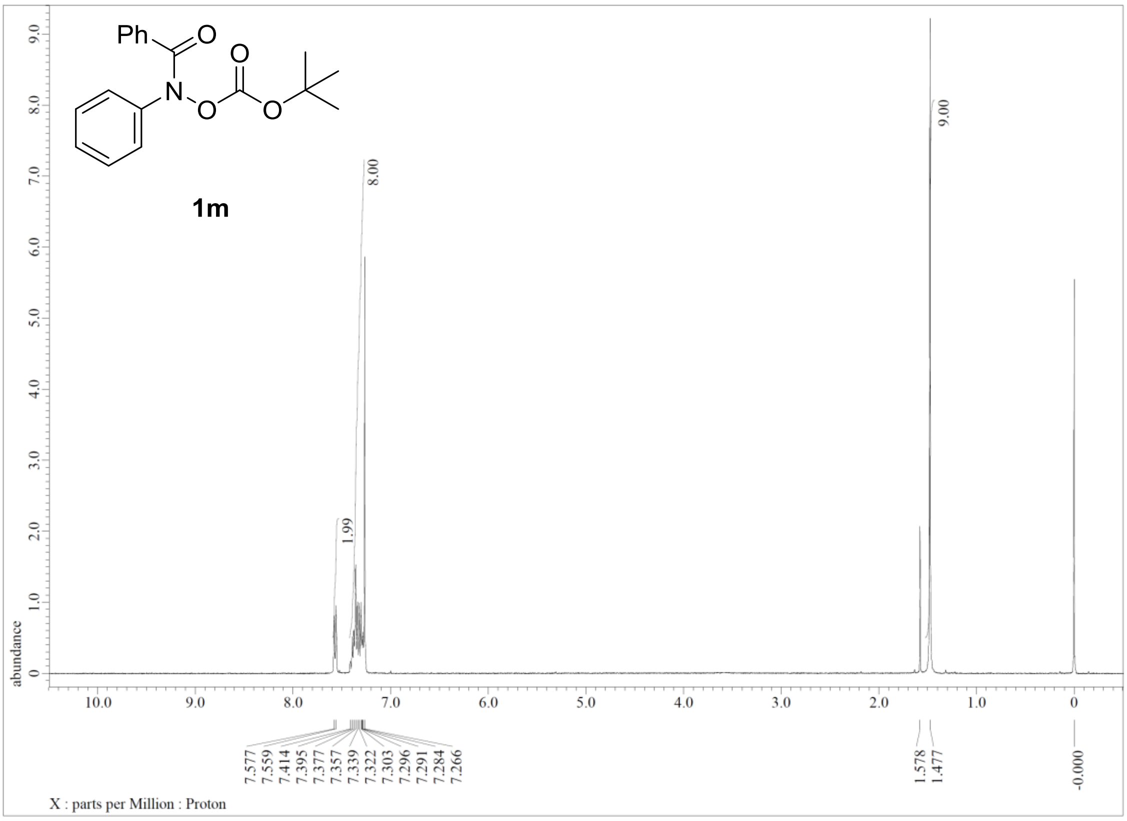

Ph
O
N
O
O
O
1m
8.00
1.99
9.00
abundance
7.577
7.559
7.414
7.395
7.377
7.357
7.339
7.322
7.303
7.296
7.291
7.284
7.266
1.578
1.477
-0.000
X : parts per Million : Proton

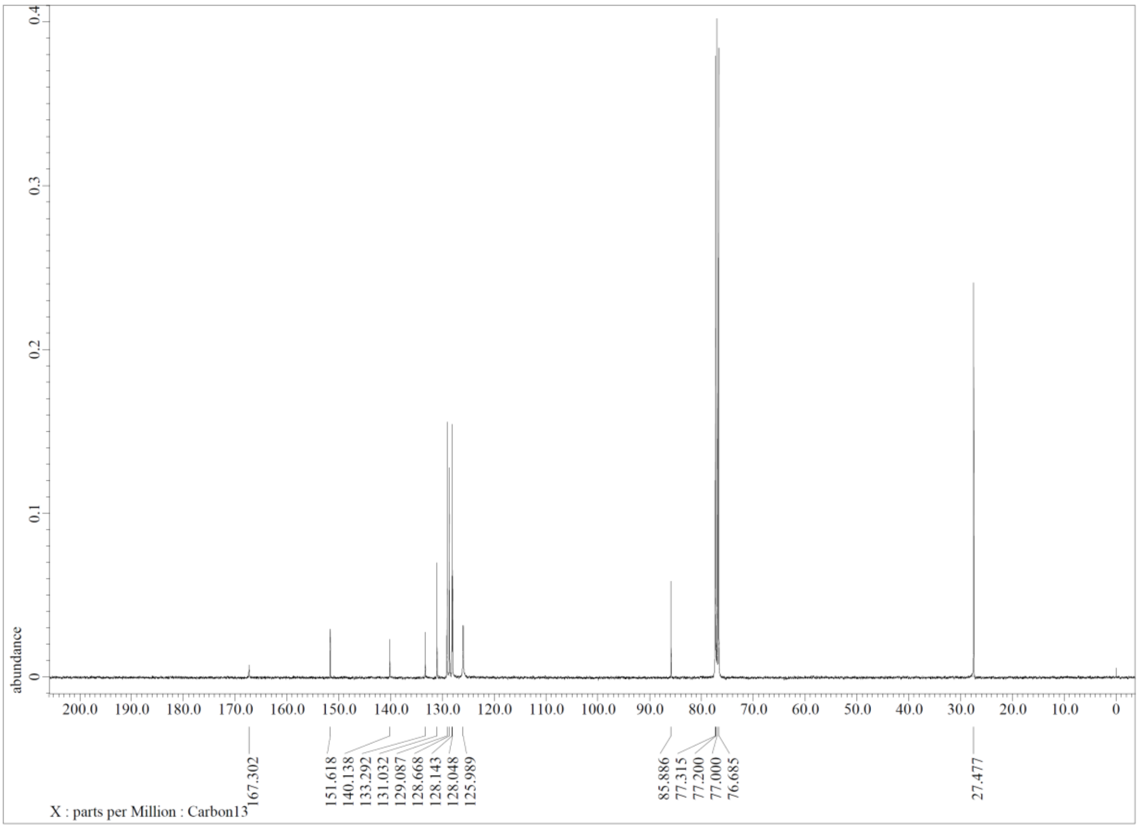

abundance
167.302
151.618
140.138
133.292
131.032
129.087
128.668
128.143
128.048
125.989
85.886
77.315
77.200
77.000
76.685
27.477
X : parts per Million : Carbon13

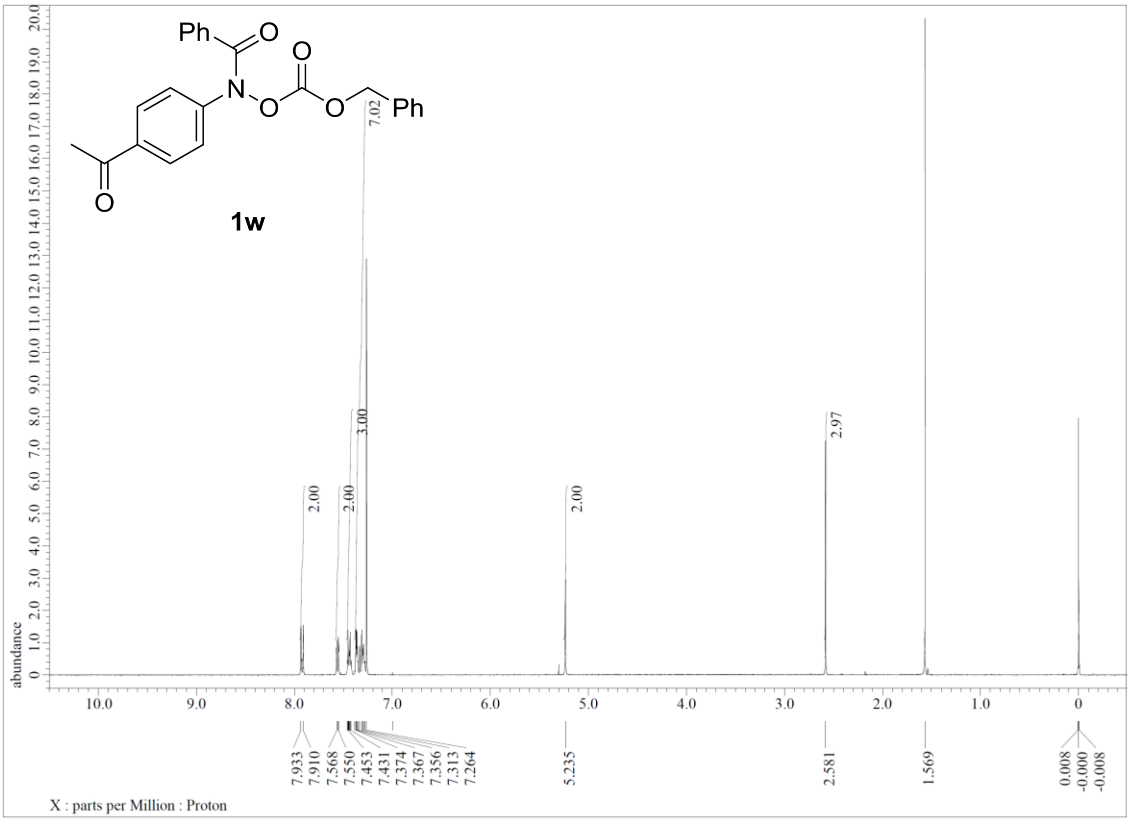
Ph
O
N
O
O
O
Ph
O
1w
abundance
X : parts per Million : Proton
7.933
7.910
7.568
7.550
7.453
7.431
7.374
7.367
7.356
7.313
7.264
5.235
2.581
1.569
0.008
-0.000
-0.008
2.00
2.00
3.00
7.02
2.00
2.97

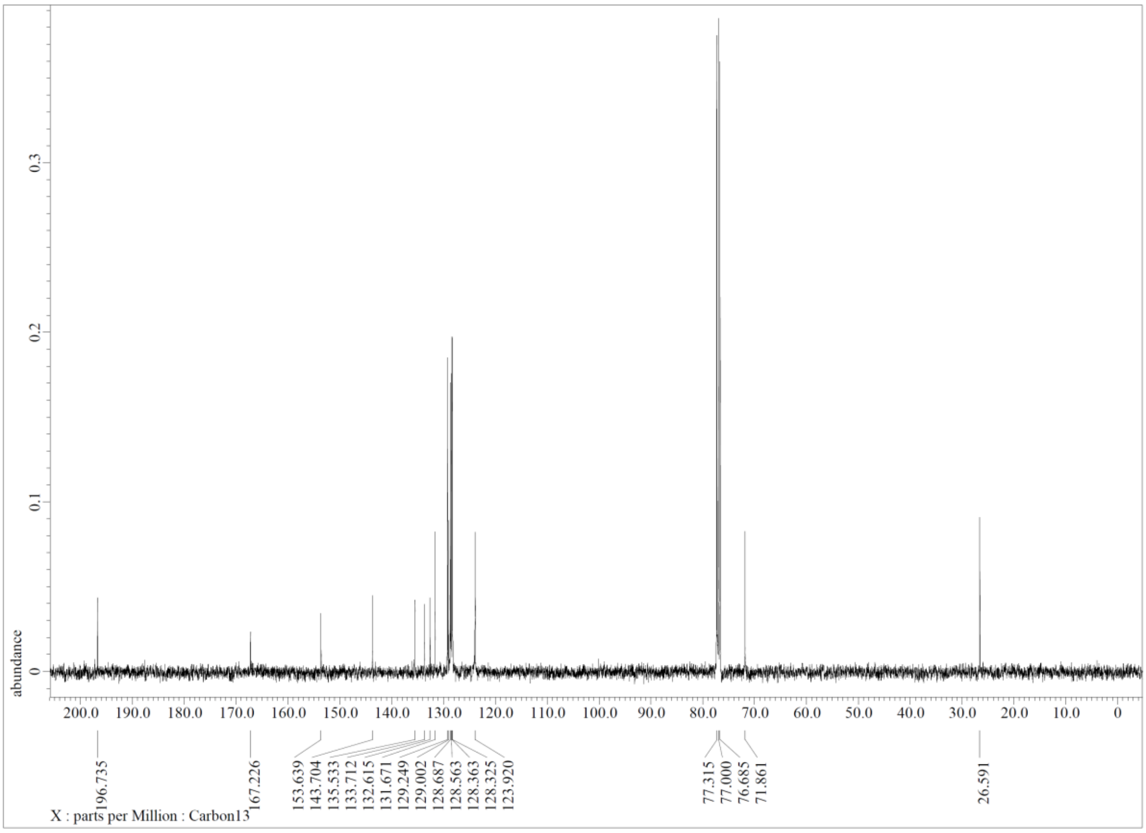
abundance
X : parts per Million : Carbon13
196.735
167.226
153.639
143.704
135.533
133.712
132.615
131.671
129.249
129.002
128.687
128.563
128.363
128.325
123.920
77.315
77.000
76.685
71.861
26.591

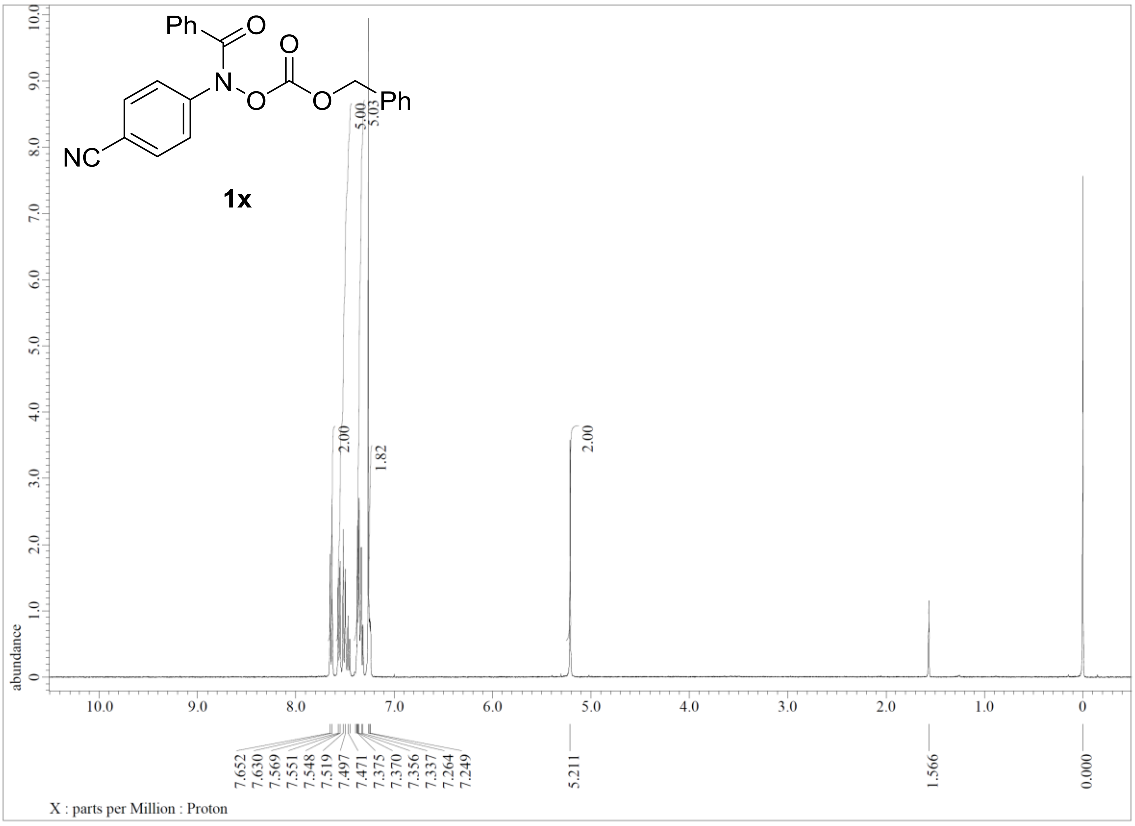
1x
abundance
X : parts per Million : Proton
7.652
7.630
7.569
7.551
7.548
7.519
7.497
7.471
7.375
7.370
7.356
7.337
7.264
7.249
5.211
1.566
0.000

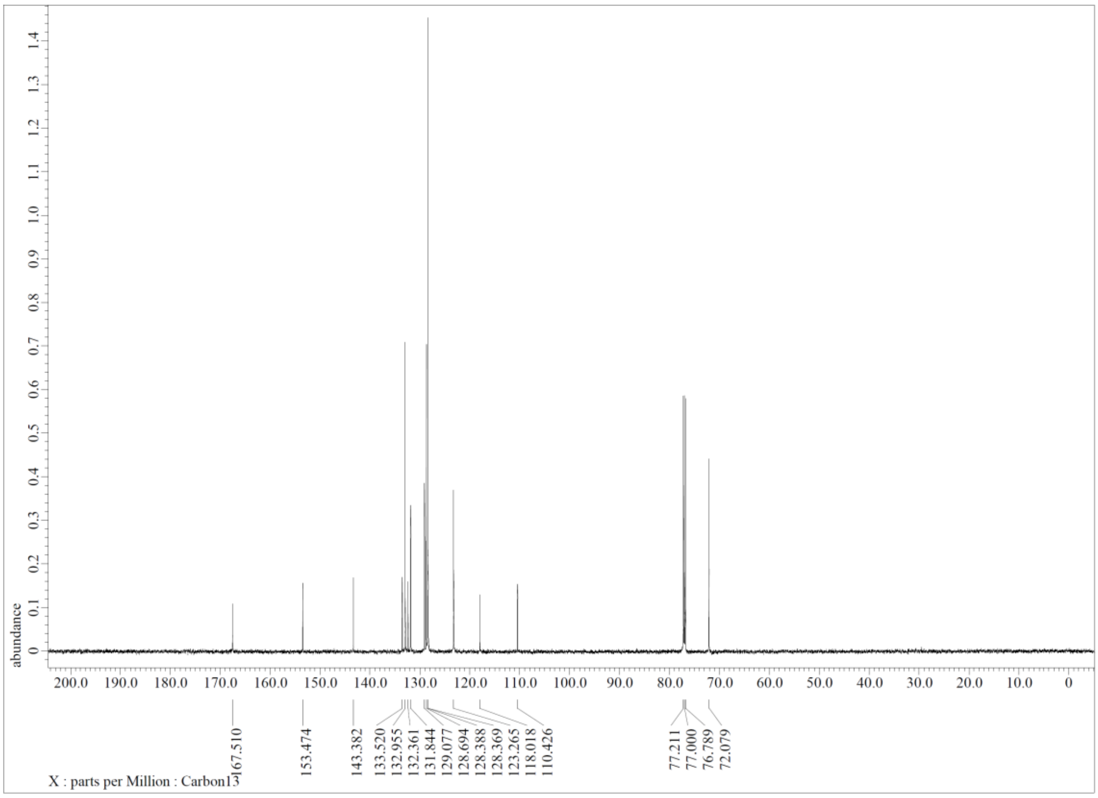
abundance
X : parts per Million : Carbon13
167.510
153.474
143.382
133.520
132.955
132.361
131.844
129.077
128.694
128.388
128.369
123.265
118.018
110.426
77.211
77.000
76.789
72.079

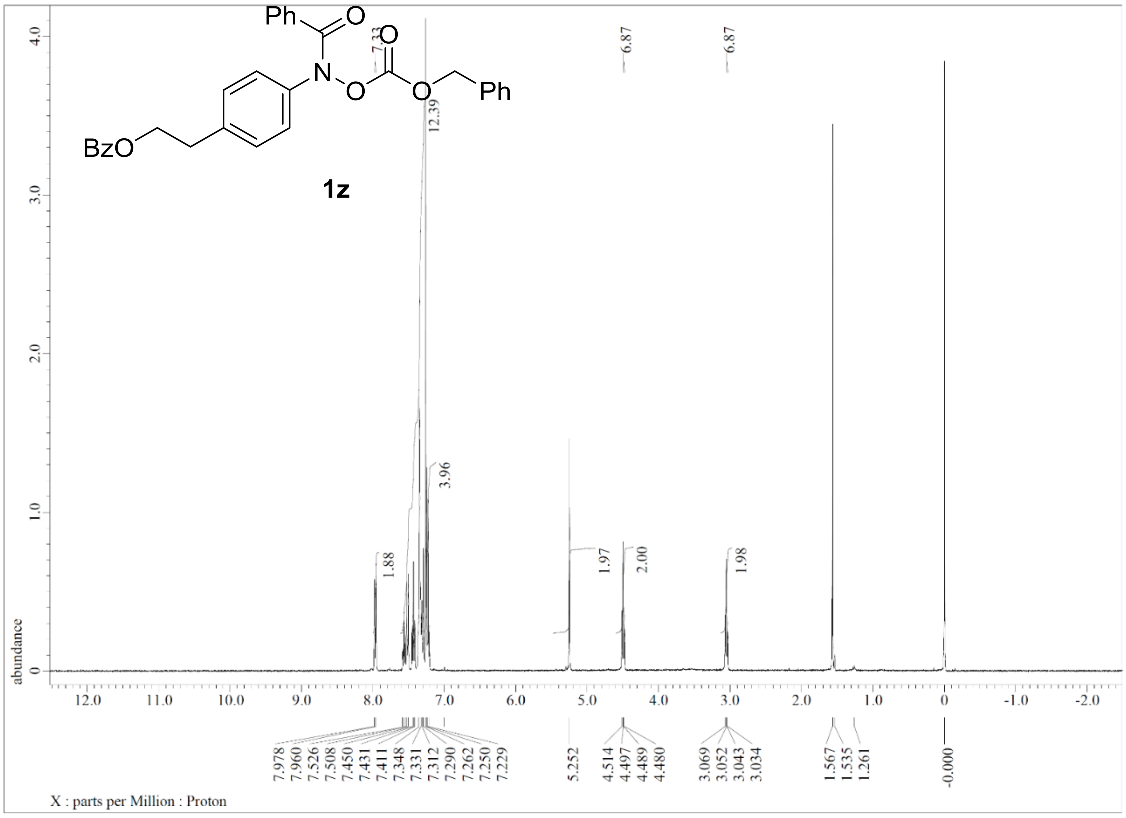
1z
BzO
Ph
N
O
Ph
abundance
X : parts per Million : Proton
7.978
7.960
7.526
7.508
7.450
7.431
7.411
7.348
7.331
7.312
7.290
7.262
7.250
7.229
5.252
4.514
4.497
4.489
4.480
3.069
3.052
3.043
3.034
1.567
1.535
1.261
-0.000
1.88
12.39
3.96
1.97
2.00
1.98
6.87
6.87

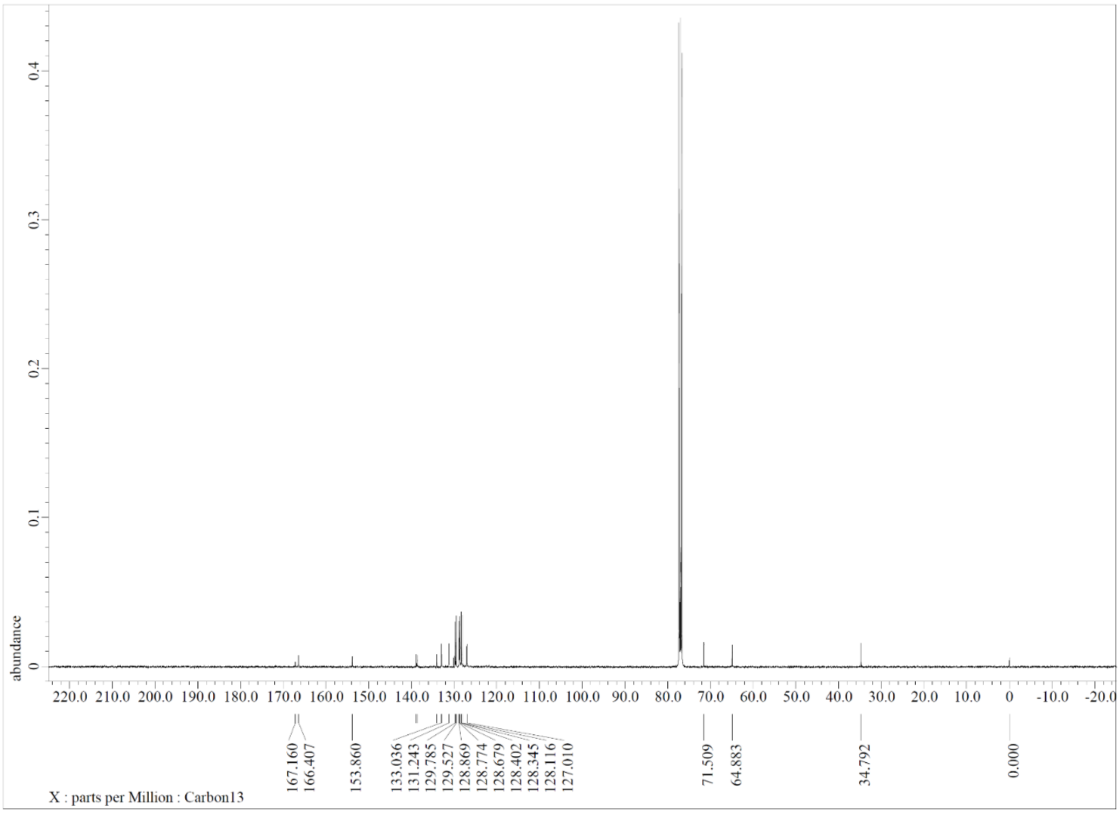
abundance
X : parts per Million : Carbon13
167.160
166.407
153.860
133.036
131.243
129.785
129.527
128.869
128.774
128.679
128.402
128.345
128.116
127.010
71.509
64.883
34.792
0.000

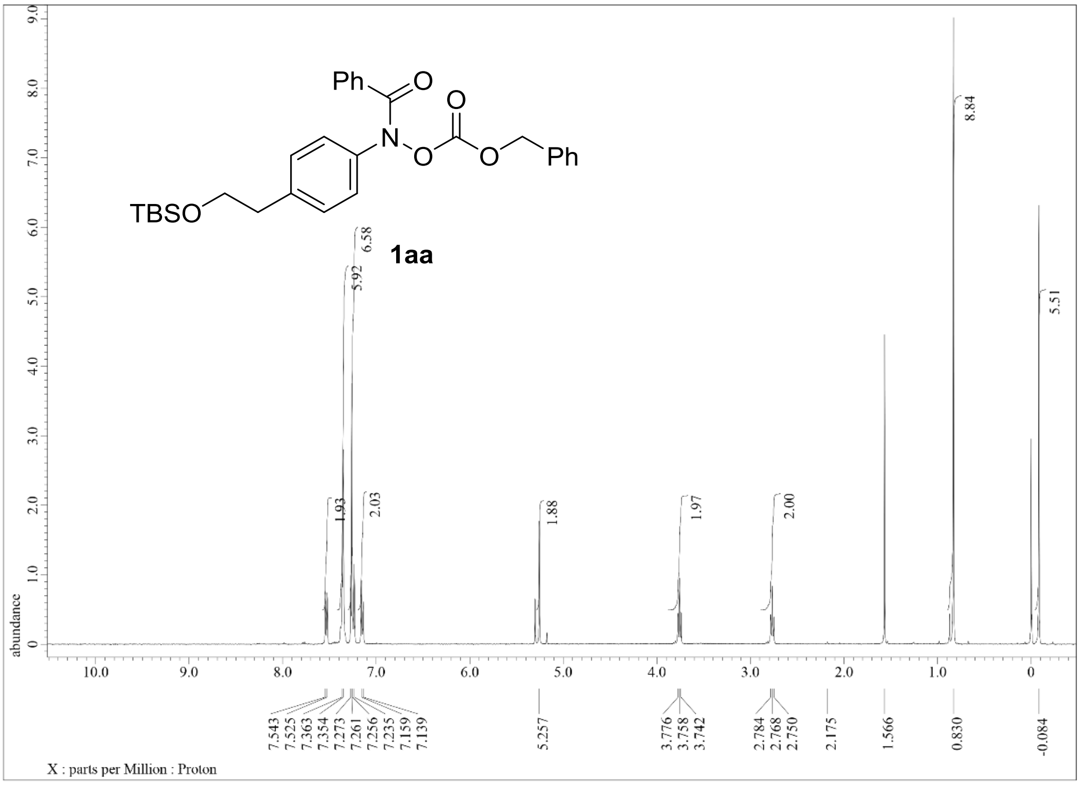
TBSO
Ph
O
N
Ph
1aa
abundance
X : parts per Million : Proton
7.543
7.525
7.363
7.354
7.273
7.261
7.256
7.235
7.159
7.139
5.257
3.776
3.758
3.742
2.784
2.768
2.750
2.175
1.566
0.830
-0.084
1.93
5.92
6.58
2.03
1.88
1.97
2.00
8.84
5.51

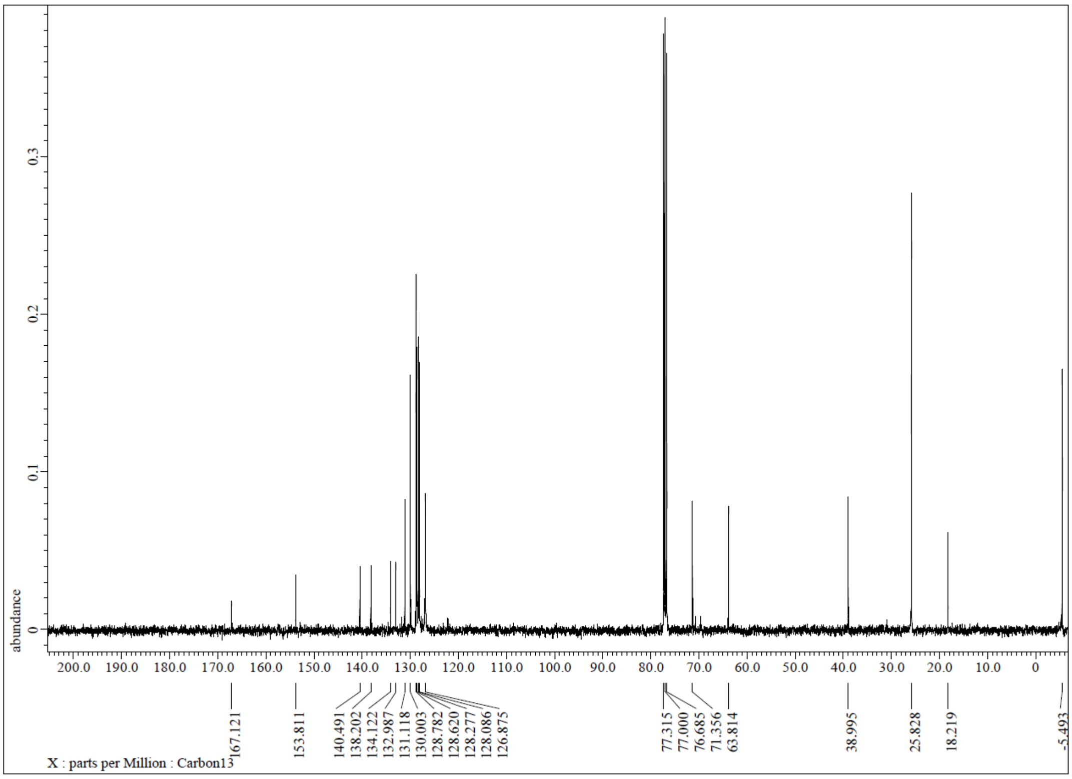
abundance
X : parts per Million : Carbon13
167.121
153.811
140.491
138.202
134.122
132.987
131.118
130.003
128.782
128.620
128.277
128.086
126.875
77.315
77.000
76.685
71.356
63.814
38.995
25.828
18.219
-5.493

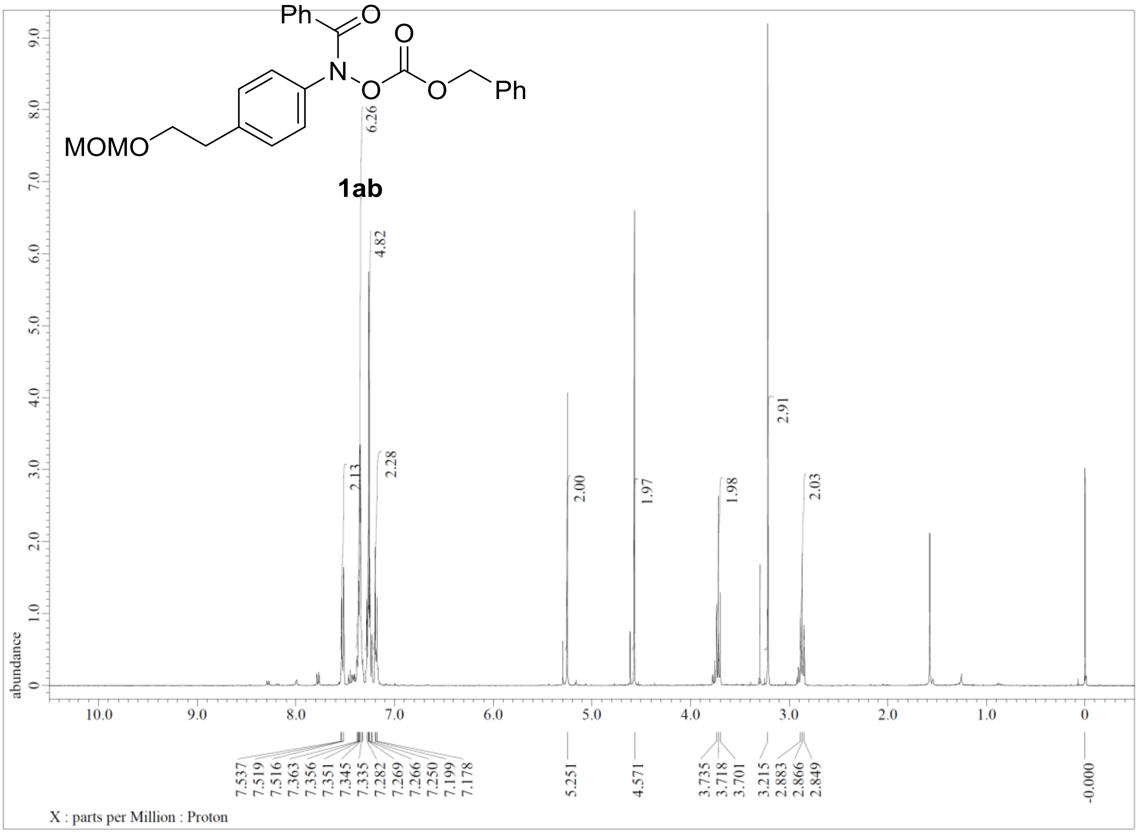
Ph
O
N
O
O
O
Ph
MOMO
1ab
X : parts per Million : Proton
abundance
6.26
4.82
2.13
2.28
2.00
1.97
1.98
2.91
2.03
7.537
7.519
7.516
7.363
7.356
7.351
7.345
7.335
7.282
7.269
7.266
7.250
7.199
7.178
5.251
4.571
3.735
3.718
3.701
3.215
2.883
2.866
2.849
-0.000

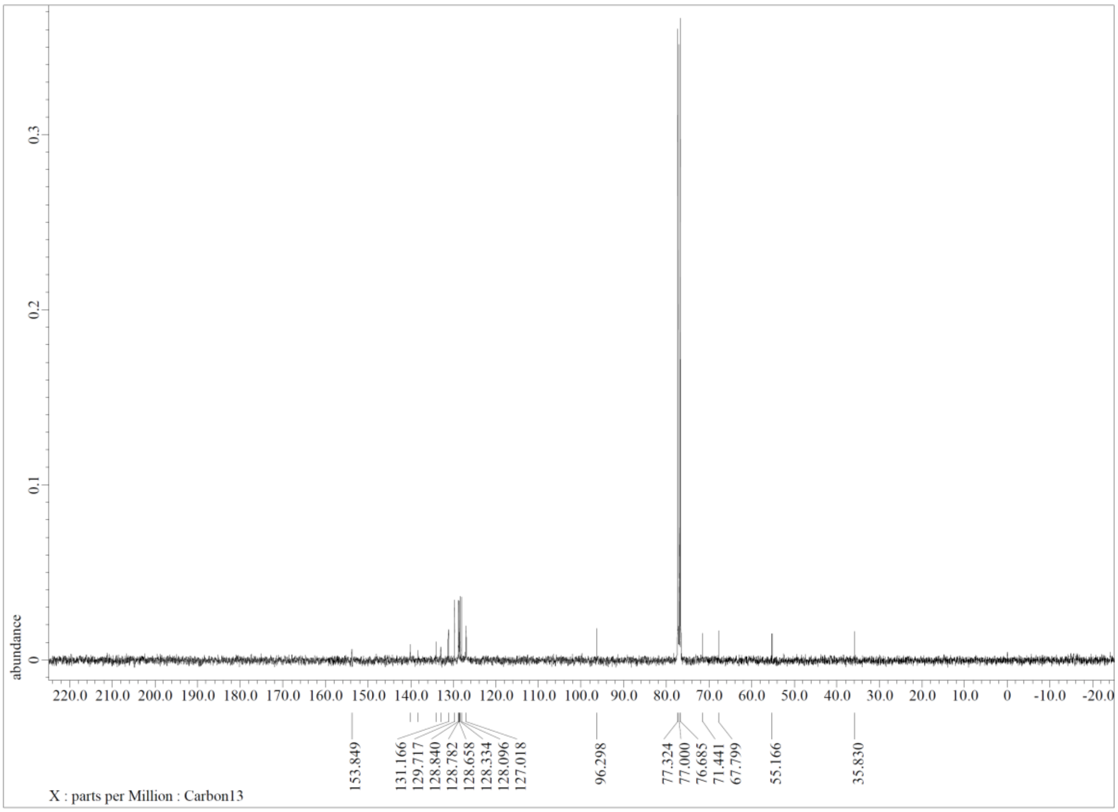
X : parts per Million : Carbon13
abundance
153.849
131.166
129.717
128.840
128.782
128.658
128.334
128.096
127.018
96.298
77.324
77.000
76.685
71.441
67.799
55.166
35.830

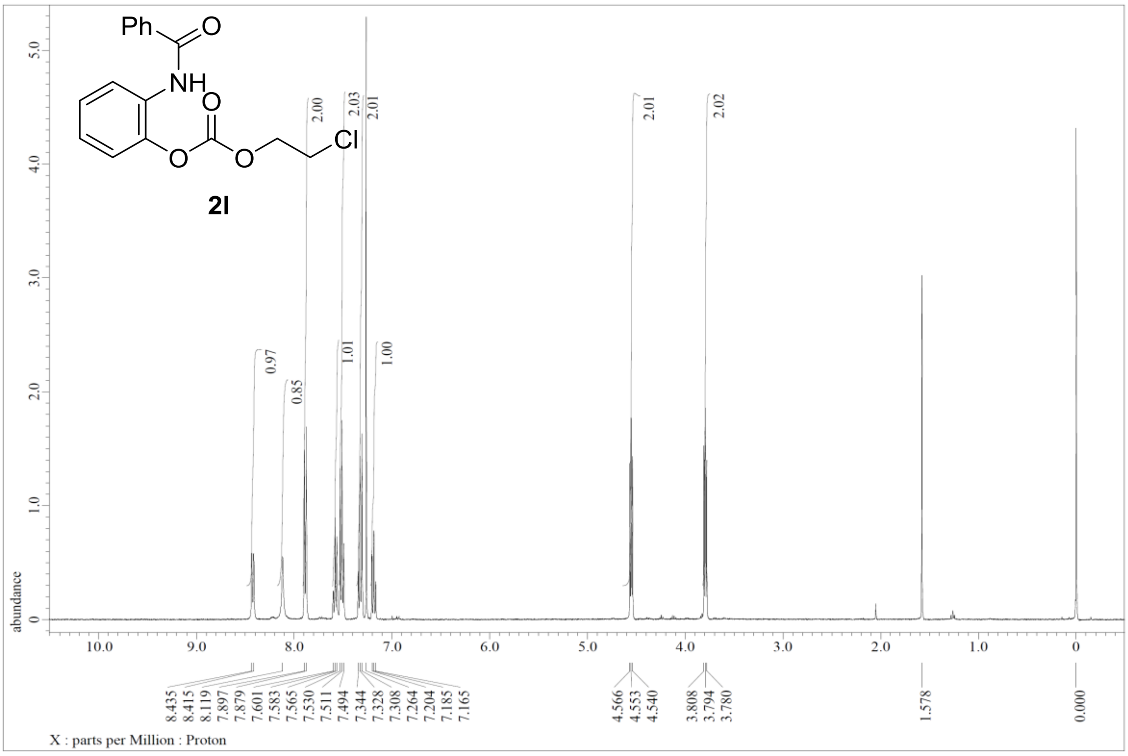
Ph
O
NH
O
O
O
Cl
2l
abundance
X : parts per Million : Proton
0.97
0.85
2.00
1.01
2.03
2.01
1.00
2.01
2.02
8.435
8.415
8.119
7.897
7.879
7.601
7.583
7.565
7.530
7.511
7.494
7.344
7.328
7.308
7.264
7.204
7.185
7.165
4.566
4.553
4.540
3.808
3.794
3.780
1.578
0.000

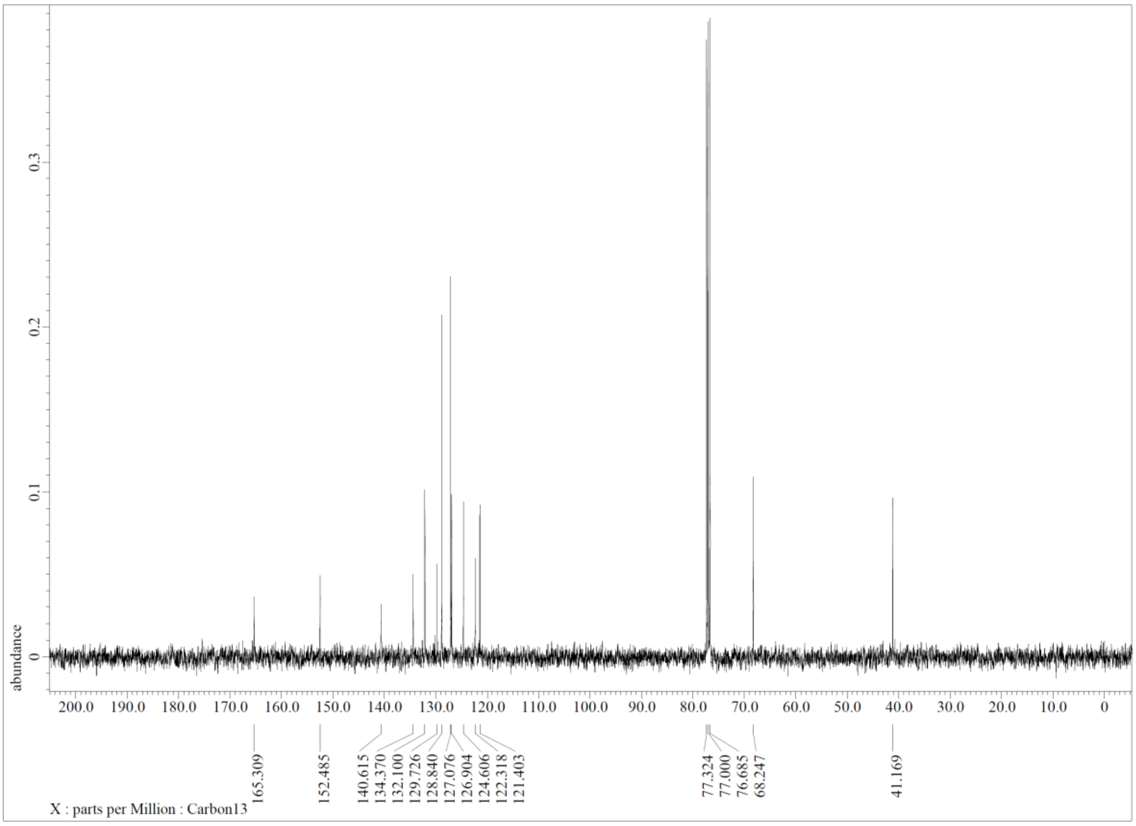
abundance
X : parts per Million : Carbon13
165.309
152.485
140.615
134.370
132.100
129.726
128.840
127.076
126.904
124.606
122.318
121.403
77.324
77.000
76.685
68.247
41.169

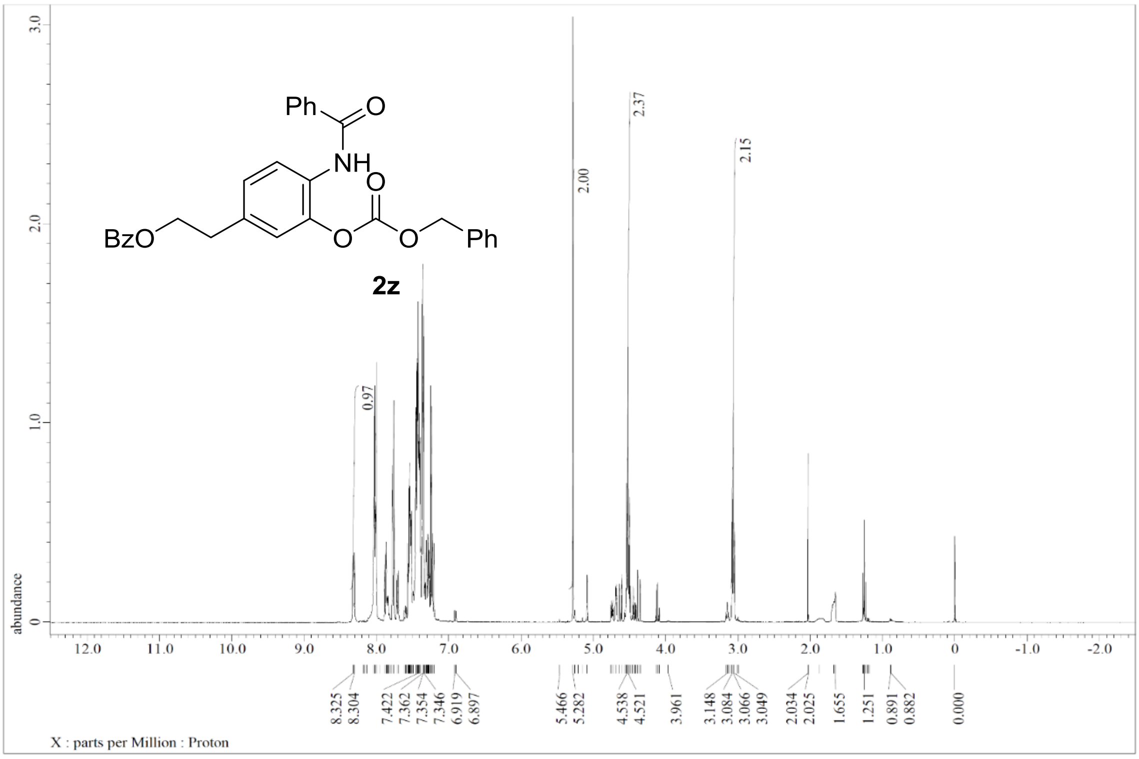
Ph
O
NH
O
BzO
O
O
Ph
2z
abundance
X : parts per Million : Proton
0.97
2.00
2.37
2.15

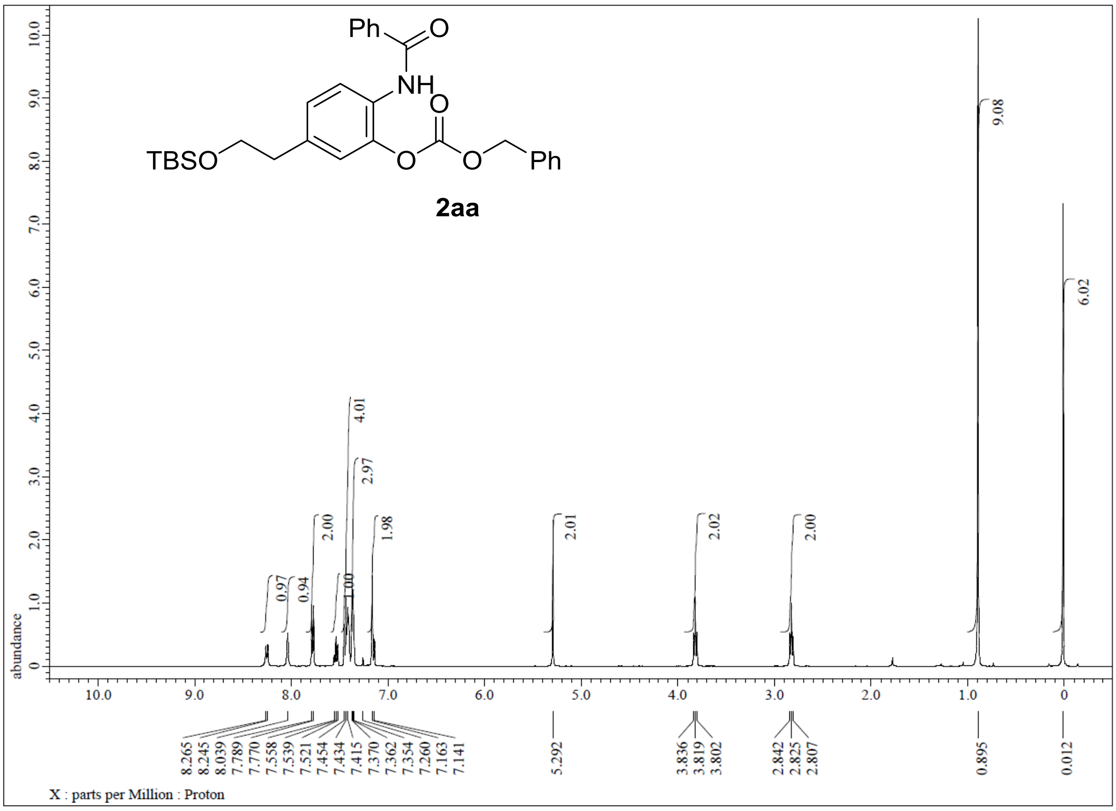
2aa
X : parts per Million : Proton
abundance

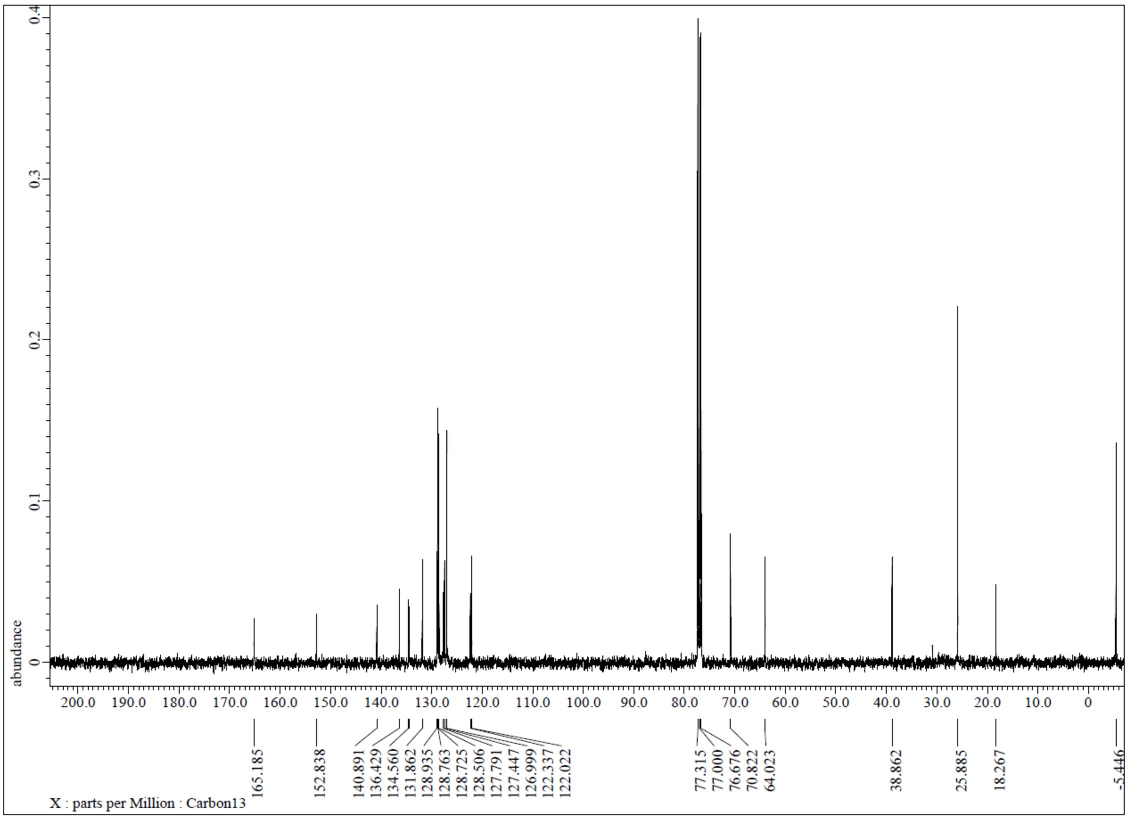
X : parts per Million : Carbon13
abundance

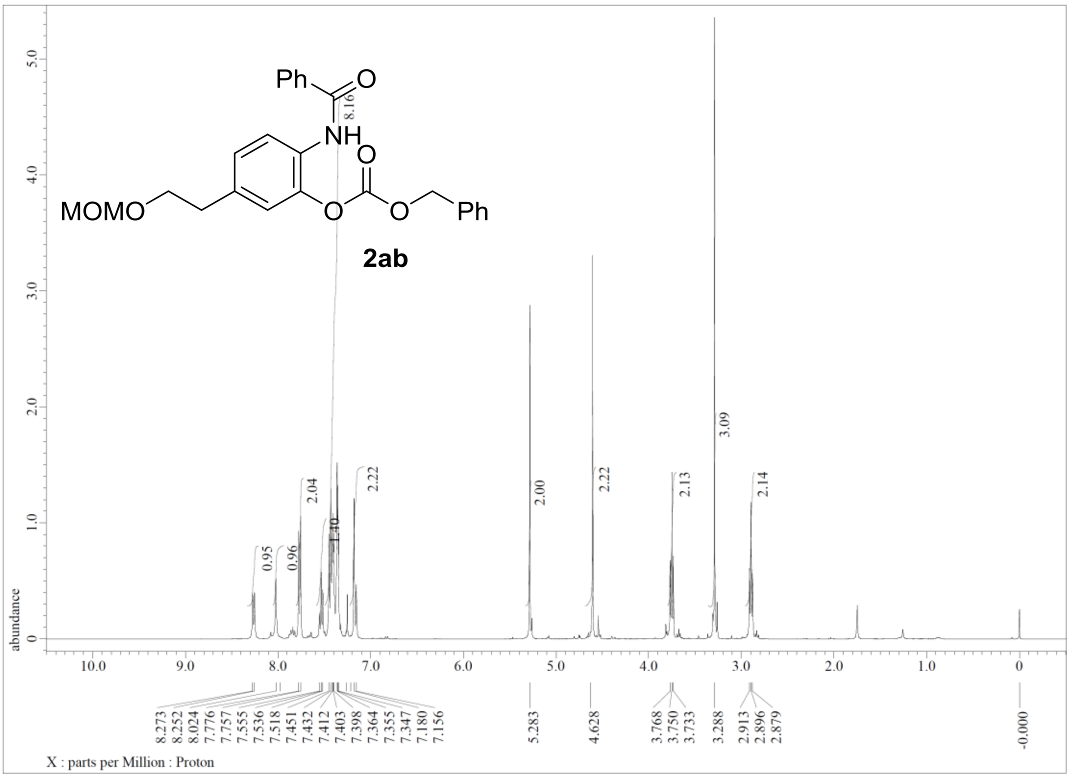
Ph
O
NH
O
MOMO
O
O
Ph
2ab
8.273
8.252
8.024
7.776
7.757
7.555
7.536
7.518
7.451
7.432
7.412
7.403
7.398
7.364
7.355
7.347
7.180
7.156
5.283
4.628
3.768
3.750
3.733
3.288
2.913
2.896
2.879
-0.000
X : parts per Million : Proton
abundance

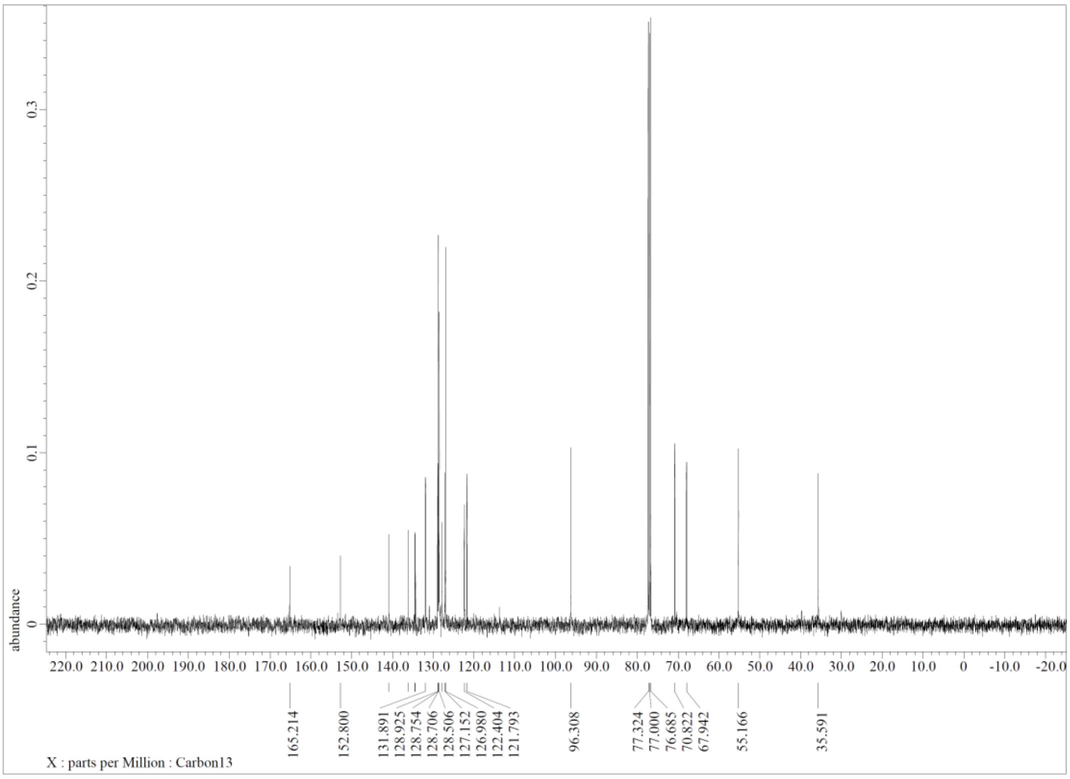
165.214
152.800
131.891
128.925
128.754
128.706
128.506
127.152
126.980
122.404
121.793
96.308
77.324
77.000
76.685
70.822
67.942
55.166
35.591
X : parts per Million : Carbon13
abundance

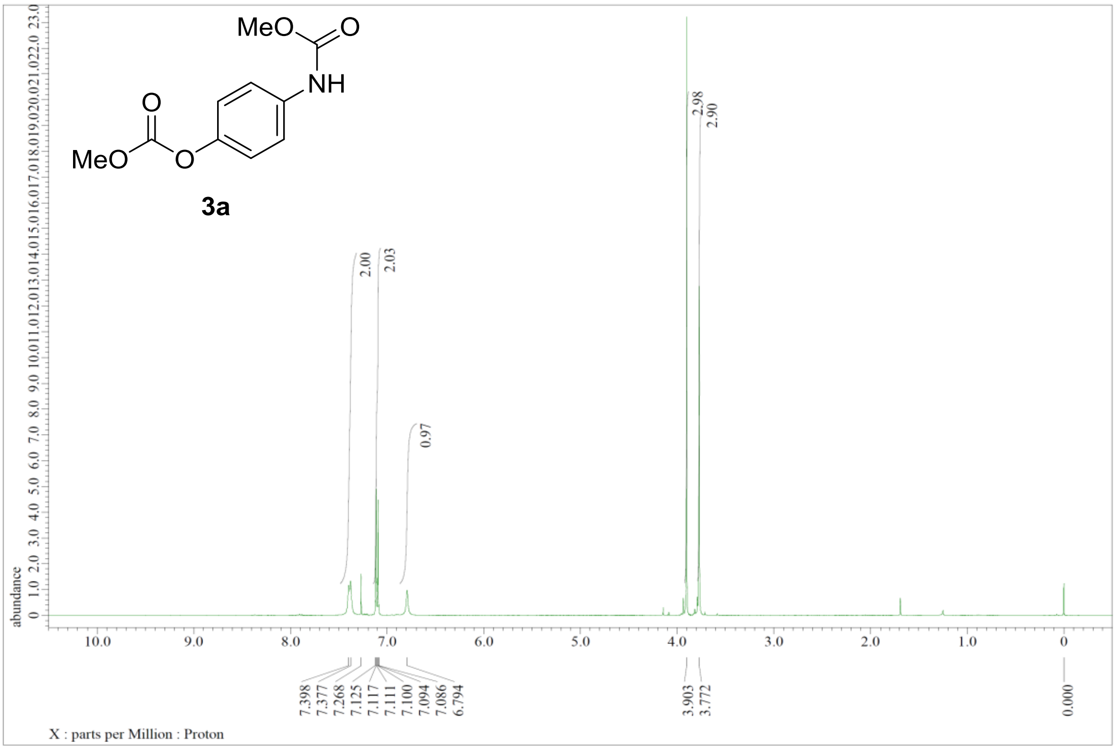

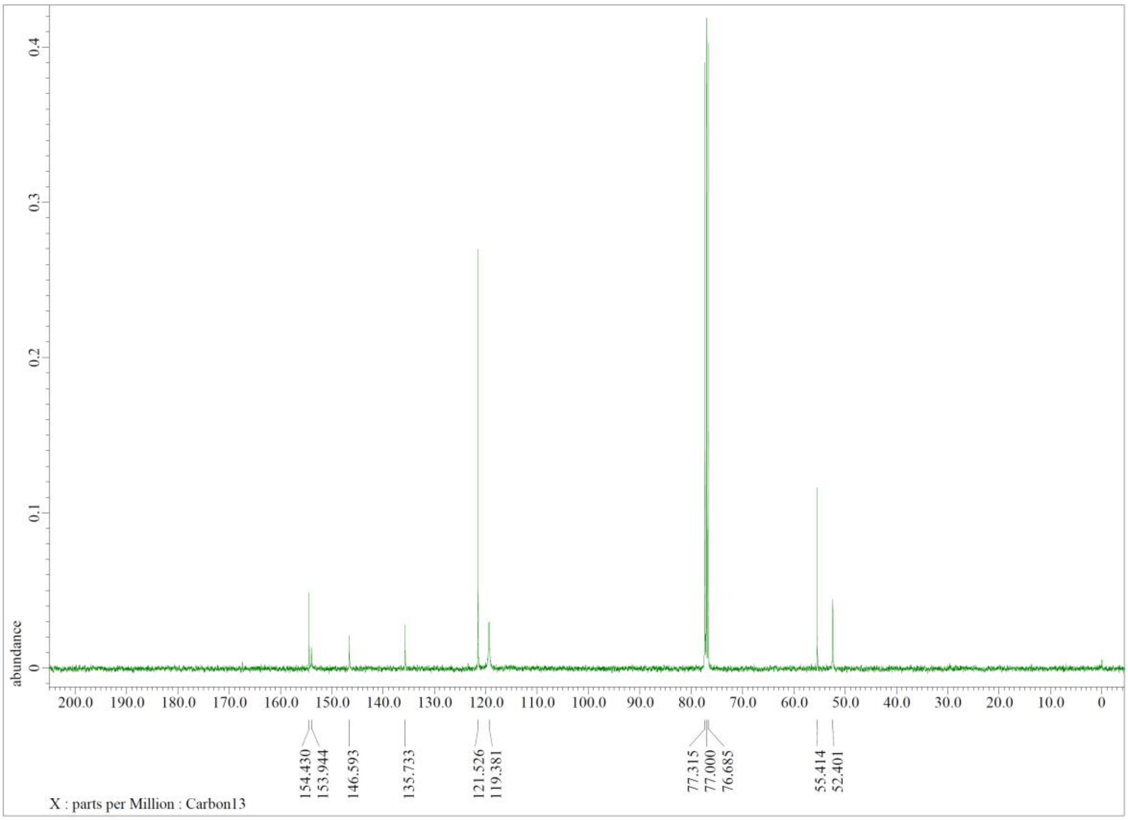

10. References

[S1] Nakamura, I.; Owada, M.; Jo, T.; Terada, M. *Org. Lett.* **2017**, *19*, 2194.

[S2] Rajendran, G.; Van Etten, R. L. *Inorg. Chem.* **1986**, *25*, 876.

[S3] Rajendran, G.; Santini, R. E.; Van Etten, R. L. *J. Am. Chem. Soc.* **1987**, *109*, 4357.
